# Intricate Proteoform Heterogeneity in the Tailpiece of IgA within and across Its Circulatory and Secretory Configurations

**DOI:** 10.1021/jacs.6c12113

**Published:** 2026-10-21

**Authors:** Weiwei Wang, Amber D. Rolland, Gestur Vidarsson, Johannes B. van Goudoever, Karli R. Reiding, Albert Bondt, Albert J. R. Heck

**Affiliations:** † Biomolecular Mass Spectrometry and Proteomics, Bijvoet Center for Biomolecular Research and Utrecht Institute for Pharmaceutical Sciences, 8125 Utrecht University, Padualaan 8, Utrecht 3584 CH, The Netherlands; ‡ Netherlands Proteomics Center, Padualaan 8, Utrecht 3584 CH, The Netherlands; § School of Pharmaceutical Science, Shanghai Jiao Tong University, 800 Dongchuan Road, Shanghai 200240, People’s Republic of China; ∥ Sanquin Research and Landsteiner Laboratory, Department of Experimental Immunohematology, 522567 Amsterdam University Medical Center, Amsterdam 1066 CX, The Netherlands; ⊥ Emma Children’s Hospital, Amsterdam Reproduction & Development Research Institute, Department of Pediatrics, Amsterdam University Medical Center, Meibergdreef 9, Amsterdam 1105 AZ, The Netherlands

## Abstract

Human immunoglobulin A (IgA) naturally occurs in multiple molecular forms—monomers, dimers, and even higher-order polymers—that are differentially distributed across serum, breast milk, and mucosal surfaces. The C-terminal tailpiece of IgA is essential to its polymerization mediated by the joining chain (J-chain) and thus also an important determinant of its extensive structural heterogeneity, yet its features remain somewhat poorly defined. Here, we used HILIC-LC-MS/MS-based glycopeptide analysis to characterize the *N*-glycan structures, disulfide linkages, and C-terminal amino acid sequence variants within the IgA tailpiece across the different molecular configurations of endogenous IgA. We find extensive correlation between these post-translational modifications; i.e., variations in tailpiece Asn459 *N*-glycosylation, redox status and disulfide connectivity of Cys471, and the presence or absence of C-terminal Tyr472 are intrinsically interconnected. When Cys471 was disulfide-bound to the J-chain, Asn459 glycans showed broader structural heterogeneity and retained more high-mannose structures, whereas free Cys471 tailpieces mainly carried mature, sialylated complex-type glycans. Serum IgA and milk SIgA displayed distinct tailpiece signatures, including differences in *N*-glycan maturation and processing of the ultimate C-terminal Tyr472 residue. Together, these findings define biofluid-specific molecular forms of IgA and highlight the remarkable structural diversity of the human IgA tailpiece. Overall, our results advance the understanding of the IgA structure and could offer a much-needed molecular basis to assist future bioengineering of IgA-based therapeutics.

## Graphical abstract

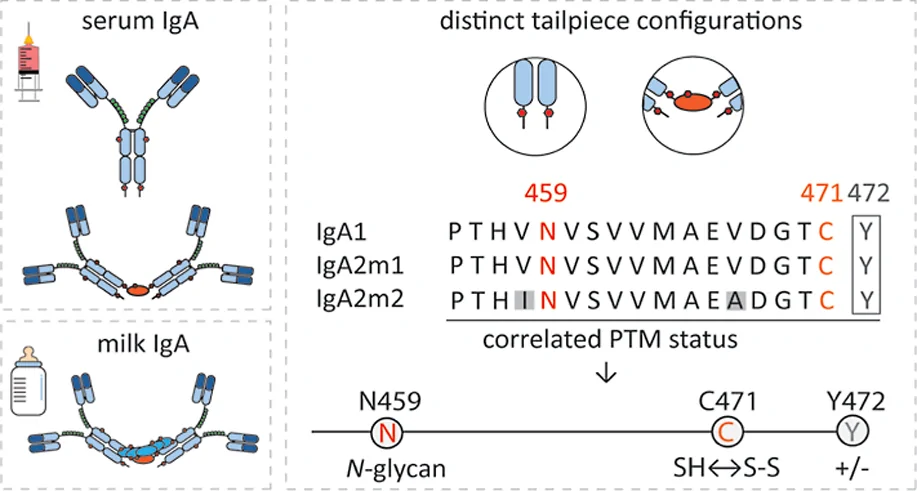

## Introduction

Human immunoglobulin A (IgA), which comprises two subclasses (IgA1 and IgA2), is the second-most prevalent serum immunoglobulin after IgG and is the predominant antibody class found in external secretions and at mucosal surfaces (Figure 1A,B).^1^ Overall, this makes IgA the most abundant antibody present in the human body, and a considerable amount of energy is dedicated to near-constant synthesis of IgA in large quantities, with daily estimates of production surpassing that of all other immunoglobulin classes combined.^2,3^

**Figure 1:**
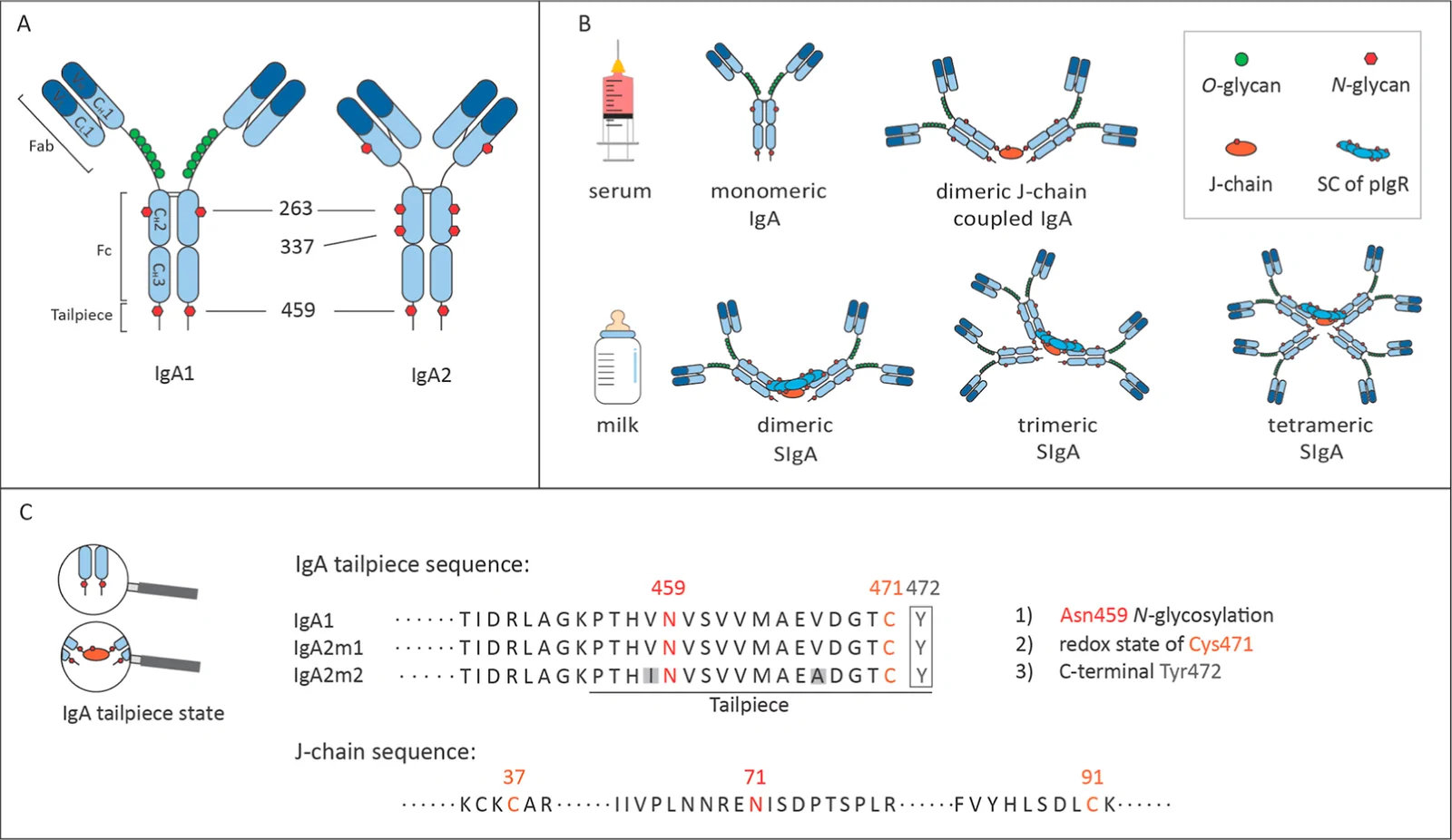
Molecular promiscuity and proteoform landscape of human IgA. (A) *N*-glycosylation sites of human IgA. Human IgA comprises two subclasses, IgA1 and IgA2, which differ in their glycosylation patterns. IgA1 carries nine putative *O*-glycosylation sites in the hinge region and contains two *N*-glycosylation sites in the Fc region. IgA2 (including the two major allotypes IgA2m1 and IgA2m2) contains three conserved *N*-glycosylation sites in the Fc region and, depending on the allotype, carries one (IgA2m1) or two (IgA2m2, not shown) additional *N*-glycosylation sites in the Fab region. In addition, the J-chain contains one *N*-glycosylation site at Asn71, and the secretory component (pIgR) contains seven *N*-glycosylation sites. (B) Molecular forms of IgA in serum and milk. In human serum, IgA is present predominantly as monomeric IgA (mIgA, ∼83–97%), with a smaller fraction occurring as J-chain-coupled dimeric IgA (dIgA, ∼4–17%).^4^ In milk, IgA is mainly present as secretory IgA (SIgA), which is predominantly dimeric but also includes higher-order polymers such as trimers and tetramers. (C) The C-terminal tailpiece of IgA is required for IgA polymerization and contains three closely positioned structural features: an *N*-glycosylation site at Asn459, a reactive thiol at Cys471 that can adopt multiple redox states, and a variable C-terminus ending at either Cys471 or Tyr472. In dIgA and dimeric SIgA, two IgA monomers are linked by the J-chain, resulting in a total of four tailpieces per assembly. Tailpiece Cys471 can form intermolecular disulfide bonds with either Cys37 or Cys91 of the J-chain, thereby coupling IgA to the J-chain. The tailpiece sequences of IgA1 and IgA2m1 are identical and highly similar to those of IgA2m2, with the same three defining structural features: Asn459 *N*-glycosylation, Cys471 redox/connectivity state, and the presence or absence of the C-terminal Tyr472.

In contrast to IgG, human IgA is found in both monomeric and polymeric forms (Figure 1B). Polymeric assemblies of IgA are primarily dimeric^5^ but also include larger structures possibly up to pentamers, and this polymerization is enabled by the 18-residue C-terminal peptide extension, known as the tailpiece. As illustrated in Figure 1C, the IgA tailpiece contains three noteworthy features in this regard: an *N*-glycosylation site at Asn459,^6–8^ a penultimate cysteine residue at Cys471,^9^ and a C-terminal tyrosine at Tyr472.^10–12^ (A detailed overview of the IgA *N*-glycosylation sites and of the amino acid numbering convention used is provided in Table S1.) Cys471 is supposedly “free” in monomeric IgA (mIgA)^13,14^ but enables covalent association with the J-chain via a disulfide bridge, leading to the formation of J-chain-coupled dimeric IgA (dIgA).^1,15^ This dIgA, as well as other J-chain-containing polymeric IgA assemblies (pIgA), can be transported across mucosal epithelial cells by binding to the polymeric immunoglobulin receptor (pIgR). At the end of this pIgR-mediated transcytosis process, the extracellular domain of pIgR referred to as the secretory component (SC) remains attached to IgA, thereby forming secretory IgA (SIgA).

SIgA is the predominant IgA species in secretions such as breast milk and at mucosal surfaces, where it serves as the first line of defense against invading pathogens.^16^ The oligomeric state of SIgA is dictated by the polymeric J-coupled IgA structure that SC binds, and SIgA is therefore predominantly dimeric, with a smaller fraction present as higher-order polymers, including trimers, tetramers, and possibly pentamers (Figure 1B).^17–19^ By contrast, IgA in human serum consists predominantly of mIgA, although a minor fraction (7–30%) is present as J-coupled dIgA (Figure 1B).^4,20–22^ All these distinct features of IgA—i.e., the co-occurring molecular configurations, subclasses, glycosylation, and tailpiece sequences—are summarized in Figure 1.

The structural characteristics of IgA, particularly its polymerization, which is enabled by the C-terminal tailpiece, have attracted quite some interest over the years.^12^ Previous studies have identified the minimum components required for the formation of polymeric IgA, which include the presence of the C-terminal tailpiece with an *N*-glycan attached to Asn459 and the penultimate cysteine residue (Cys471) responsible for covalent association to the J-chain via disulfide linkages. The J-chain must also be present with its two respective cysteine residues (Cys37 and Cys91, Figure 1C), with glycosylation of its single site at Asn71 not strictly required for initiation of assembly but appearing instead to contribute to complex stabilization.^4^ However, most of these studies were conducted on recombinant IgA or murine IgA,^23–25^ and, as a consequence, understanding of endogenous human IgA structure and function—especially in serum and under healthy conditions—remains far from complete, with several important questions still unanswered.^26^

For example, the range of *N*-glycan structures attached to the C-terminal tailpiece at site Asn459 and/or to the J-chain’s single site at Asn71, which are amenable to the formation of J-coupled pIgA, has not been fully elucidated. *N*-glycans attached to these sites have also not been systematically compared between all distinct molecular forms of IgA present across both systemic and mucosal immune compartments. While two tailpieces are disulfide-linked to the J-chain via Cys471 in all J-coupled polymeric assemblies of IgA including secretory forms, the role of the C-terminal tailpiece—and particularly of Cys471—in monomeric IgA remains largely unexplored. Notably, Cys471 thiol reactivity has been implicated in the extracellular association of mIgA with several other proteins in serum.^11^ Moreover, whether the “free” tailpieces in mIgA can also form intrasubunit disulfides is not well known. Additionally, details of the covalent interactions involving the tailpieces in lower-order pIgA assemblies (i.e., J-coupled dimers and dimeric SIgA), which are also expected to be free, based on the cryo-EM structures of SIgA,^27^ remain unclear. Answering these questions is highly relevant not only to IgA function but also to further elucidate the detailed assembly mechanism by which J-coupled IgA polymers are formed. Finally, it has been reported that the ultimate C-terminal tyrosine residue at position 472 is sometimes missing from tailpiece-containing peptides detected in proteomic analyses,^28–32^ but its prevalence in different IgA molecular forms and physiological relevance also remain unclear.^2,29–31^ Together, these unresolved aspects point to the central questions of this work: how structural features within the IgA tailpiece are correlated within the different molecular forms in circulation and mucosal secretions, and whether there is any correlated behavior between these three post-translational modifications (i.e., *N*-glycosylation, Cys-linkage and Tyr-clipping).

To address these questions, we focused on three distinct molecular forms of IgA: human milk SIgA, serum mIgA, and serum dIgA. We aimed to delineate all three above-mentioned structural features in the C-terminal tailpiece (i.e., Asn459 *N*-glycosylation, redox state of Cys471, and presence/absence of the C-terminal Tyr472) in these distinct molecular forms of IgA. Analysis of these three features in parallel allowed us to observe correlated behavior, providing a more comprehensive picture of the full glycoproteoform landscape of human IgA.

## Results

### Experimental Design

Prior to a more detailed investigation of the IgA C-terminal tailpiece across different molecular forms, we first analyzed the general *N*-glycan signatures in the Fc domain of human IgA as present in serum and milk. The subclass and assembly distribution of IgA are particularly different in these two biological fluids, as illustrated in Figure 1B.^33,34^ SIgA (which is primarily dimeric but also includes higher-order polymers possibly up to pentamers) is the dominant form present in milk, where both IgA subclasses are roughly equally abundant. In contrast, serum IgA is dominated by mIgA with a minor population of dIgA assemblies also present (representing up to 30% of the total IgA in serum) and also by the IgA1 subclass, which typically accounts for ∼80–90% of the total IgA in serum, though the exact mIgA/dIgA and IgA1/IgA2 ratios can vary between individuals.^4^

IgA1 and IgA2 (Figure 1A) have high sequence homology but still differ with respect to their glycosylation sites, disulfide bonds, and several other structural (e.g., hinge region) and functional features. Two *N*-glycosylation sites in the Fc region, namely, Asn263 in the CH2 domain and Asn459 in the tailpiece, are conserved across both subclasses. The sequence surrounding Asn263 is identical for both subclasses, which is important for this study, as glycopeptides containing this CH2 *N*-glycosylation site therefore cannot be distinctly attributed to either IgA1 or IgA2. By contrast, the tailpiece sequence differs slightly between IgA2 allotypes (Figure 1C) despite the glycosylation site at Asn459 being conserved across all human IgA. Notably, the tailpiece sequence for one of the IgA2 allotypes (IgA2m1) is identical to that of IgA1. The IgA2 Fc region contains one additional *N*-glycosylation site (Asn337 in CH2).

*N*-Glycosylation motifs are also present in the sequences of IgA’s cofactors (J-chain and SC of pIgR, Figure 1B). These moieties are therefore specific to distinct molecular forms of IgA, including Asn71 in the J-chain, which is found only in polymeric IgA assemblies (e.g., dIgA and SIgA). We included these four glycosylation sites—i.e., Asn263, Asn337, and Asn459 in IgA and Asn71 in the J-chain—in our analysis but focused mainly on the highly abundant tailpiece sequence shared by the IgA1 subclass and the IgA2m1 allotype, as together these represent nearly all IgA molecules in serum.

### Distinctive Features of *N*-Glycosylation in Endogenous Human IgA Purified from Milk and Serum

For this study, we obtained paired serum and milk samples for four healthy female donors (Figure 2A). We characterized IgA in all eight of these samples, allowing us to also observe any potential biological variance between donors. We performed tryptic digestion of these IgA samples followed by hydrophilic interaction liquid chromatography tandem mass spectrometry (HILIC-MS/MS) analysis on the IgA- and J-chain-derived glycopeptides (Figure 2B,C) using methods previously described.^35^ Typical glycan structures found to be attached to these peptides were identified and annotated based on their high-quality MS2 spectra (see Figures S1 and S2). The site-specific IgA *N*-glycosylation profiles of the four donors’ human milk and serum IgA are shown in Figure 2D, with the distinctive *N*-glycan features found at each site (i.e., high-mannose, hybrid, and biantennary complex-type glycan structures with or without bisection) summarized in Figure 2E. These data revealed that within the same biofluid, all four individuals’ IgA exhibited highly similar glycan features at each site, in line with observations from previous studies on human IgA glycosylation.^26,36^

**Figure 2:**
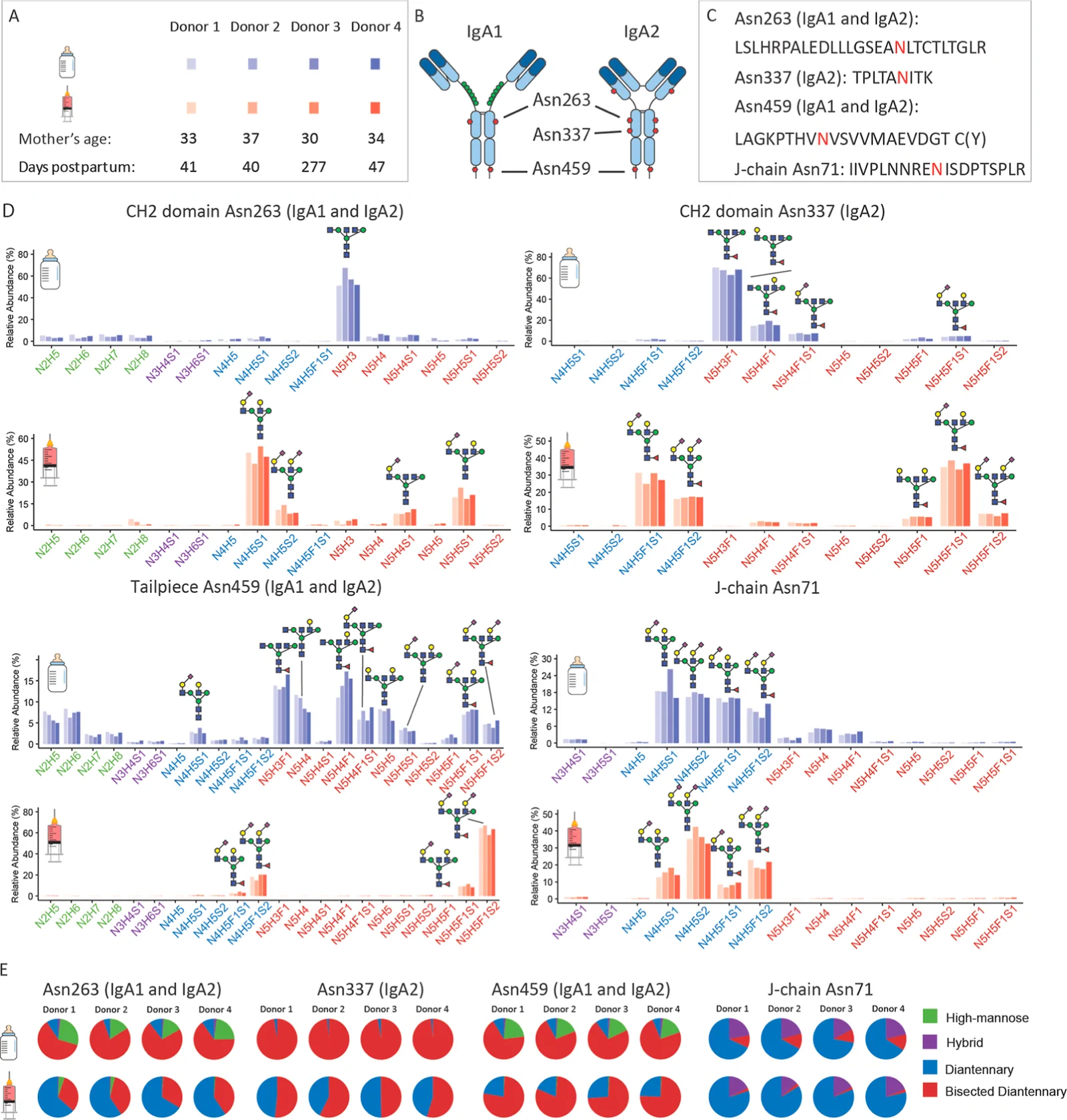
Analysis of human milk and serum IgA glycosylation features in paired donor samples. (A) Donor and sample information. Features of IgA Fc *N*-glycosylation were analyzed in paired human milk (blue) and serum (red) samples collected from four healthy female donors (Donors 1–4) ranging in age from 30 to 37 years old. Samples were collected ∼6–8 weeks after delivery, with the exception of Donor 3, as indicated by the age of each infant or number of days into the postpartum period. (B) Schematic overview and residue positions of the three *N*-glycosylation sites located in the Fc domain of human IgA. (C) Tryptic glycopeptides corresponding to the four IgA Fc and J-chain *N*-glycosylation sites that were detected in the paired milk/serum samples of Donors 1–4 and used to determine site-specific glycosylation profiles. Of note, both Asn263 and Asn459 are conserved across human IgA, and glycopeptides containing the former site are indistinguishable between all IgA1 and IgA2 variants. For Asn459, the analysis focused on the high-abundance tailpiece glycopeptide sequence shared by IgA1 and IgA2m1, whereas IgA2m2-specific tailpiece glycopeptides were not considered due to their low expected abundance. (D) Site-specific *N*-glycosylation profiles of IgA in matched milk (top, blue) and serum (bottom, red) samples of Donors 1–4, with each bar representing an individual donor. Relative abundances were determined for each sample based on quantified peak areas of all detected glycopeptides for the same site and are plotted for each different glycan composition, denoted by the number of *N*-acetylhexosamines (N), hexoses (H), fucoses (F), and *N*-acetylneuraminic acids (S). Text color indicates glycan classification into one of four types according to the color scheme shown in (E). In general, broad consensus is observed within the same sample type between donors, but features of *N*-glycosylation are highly distinct between secretory and circulatory IgA as well as across different sites within the same biofluid. (E) Overview of the distinctive features of *N*-glycosylation at each site in secretory and circulatory IgA for Donors 1–4. Each glycan was categorized based on composition as high-mannose, hybrid, or biantennary nonbisected or bisected, with the cumulative relative abundances of each type depicted in these summary pie charts according to the color scheme shown in the legend at right.

By contrast, substantial differences in the *N*-glycan signatures of IgA from milk versus serum were revealed by this comparative analysis. In the CH2 domain at Asn263, milk SIgA predominantly harbored bisected biantennary glycans (exemplified by N5H3) with low levels of sialylation and fucosylation (Figure 2D). Milk SIgA also carried mainly bisected biantennary glycans in the CH2 domain at Asn337, but glycans attached to this IgA2 subclass-specific site was almost exclusively decorated with fucose and had higher levels of galactose and sialic acid (Figure 2E). This contrasts the sialylated biantennary glycans with and without bisecting GlcNAc observed at both CH2 sites in serum IgA (Figure 2D). With respect to the tailpiece site at Asn459, milk SIgA exhibited very diverse glycan profiles, spanning from immature high-mannose to fully processed, sialylated biantennary complex-type structures, while in contrast, serum IgA tailpieces exclusively displayed fully matured sialylated nonbisected and bisected biantennary glycans.

Intriguingly, the J-chain, present in serum dIgA and milk SIgA, exhibited highly similar *N*-glycosylation patterns in both circulatory and mucosal IgA-associated contexts (Figures 2D and 2E). In both cases, all four donors’ J-chain Asn71 glycan profiles were dominated by biantennary sialylated structures. Only subtle differences were observed, namely that the milk SIgA glycan profiles contained relatively larger—but still overall minor—contributions from bisected biantennary glycans (around 10%) compared to serum (around 2%, Figure 2E).

In these glycoproteomics experiments, we also obtained data on the presence/absence of the C-terminal tyrosine (Tyr472) residue in the IgA tailpiece, revealing a distinct, substantial difference in Tyr-clipping frequency between milk and serum. In addition to the expected tryptic peptide containing the full-length tailpiece sequence terminating in Tyr472 (LAGKPTHVNVSVVMAEVDGTCY), a second peptide containing a truncated tailpiece sequence missing the C-terminal tyrosine residue and ending instead with Cys471 (LAGKPTHVNVSVVMAEVDGTC) was also observed. These tailpiece sequence variants are hereafter referred to as Tyr472+ and Tyr472–, respectively. Effectively, all IgA molecules purified from the human milk of Donors 1–4 retained the C-terminal Tyr472, with 99–100% of all tailpiece-containing peptides corresponding to Tyr472+ (Figure S3). In contrast, only a minority (∼25–42%) of the tailpieces in the serum-derived IgA of Donors 1–4 included the Tyr472 residue, while the majority (∼58–75%) were truncated with Cys471 at the C-terminus, as shown in Figure S3. These findings are in line with the observations reported by Plomp et al., where tailpiece truncation was prevalent in IgA purified from plasma but much less abundant in salivary (secretory) IgA.^37^

### Serum mIgA and dIgA Exhibit Highly Similar Glycosylation Profiles, Except for the Tailpiece

Given that the J-chain is exclusively associated with dimeric IgA in serum and with (predominantly dimeric) SIgA in milk via disulfide bridges to two tailpiece Cys471 residues, these observations raised the intriguing question of whether serum dIgA and milk SIgA have similar tailpiece *N*-glycosylation signatures. Although the Asn459 glycosylation profiles were strikingly different between the paired milk and serum samples of donors 1–4 (Figure 2D), any potential overlap would likely be obscured given that J-coupled IgA dimers are far less abundant than monomers in serum.^4^ It is therefore likely that the distinct serum IgA tailpiece *N*-glycan signatures shown in Figure 2D largely reflect monomers. Accordingly, next, we aimed to determine whether serum mIgA and dIgA exhibit distinct *N*-glycosylation signatures.

Answering these questions requires co-occurring mIgA and dIgA assemblies in serum to be separated. We therefore affinity-purified total circulatory IgA (i.e., all molecular forms and subclasses) from two healthy donor sera (Donors 5 and 6, Figure 3A) and then used single-molecule mass photometry (MP) to determine the overall assembly composition of IgA in these two samples.^4,38^ As the annotated MP mass histograms in Figure 3B show, mIgA (∼160 kDa) and dIgA (∼340 kDa) assemblies co-occurred in each donor’s circulation but with differing relative abundances, in line with what we have previously reported.^4^ With roughly 8% of the total circulatory IgA present in J-coupled dimeric assemblies (Figure 3B), Donor 6’s serum IgA assembly distribution was consistent with typically reported “average” values (i.e., with dIgA representing ∼10% of the total serum IgA). By contrast, dIgA was considerably elevated in the serum of Donor 5, where this molecular form accounted for ∼21% of the total IgA, thus representing a more abundant endogenous source of serum-derived dIgA. Subsequently, we used size exclusion chromatography (SEC) to separate mIgA and dIgA, as previously described.^4^ Each SEC fraction containing either dIgA or mIgA was then subjected to tryptic digestion followed by HILIC-LC-MS/MS analysis of the generated glycopeptides to characterize their site-specific *N*-glycosylation features and presence of the C-terminal tyrosine.

**Figure 3:**
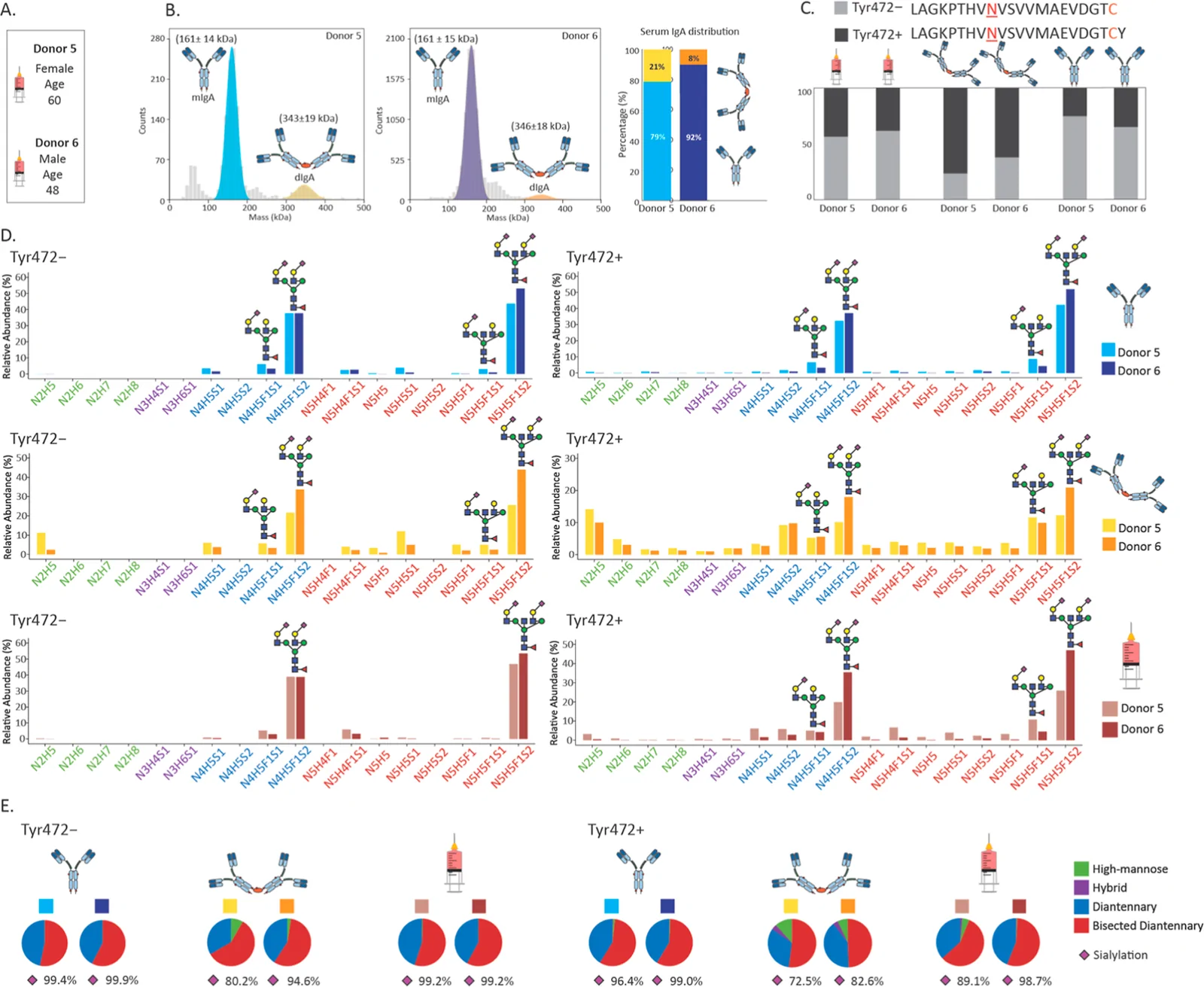
Comparative analysis of *N*-glycosylation and Tyr clipping in serum monomeric and J-chain-coupled dimeric IgA. (A) IgA was purified from the serum/plasma of two additional healthy individuals identified as Donor 5 (female, age 60) and Donor 6 (male, age 48). Each donor’s monomeric and J-chain-coupled dimeric assemblies of circulatory IgA were then separated by size exclusion chromatography. (B) Mass photometry revealed monomeric IgA (∼160 kDa) was predominant in these samples as expected. In Donor 6, slightly less than 10% of the total circulatory IgA was present in J-coupled dimeric assemblies, while in Donor 5, the dIgA accounted for greater than 20% of all IgA in circulation. (C) In all three samples analyzed for each donor (i.e., the SEC-separated mIgA and dIgA, and the unfractionated total circulatory IgA in the originating serum sample), two distinct IgA1 tailpiece sequence variants—with (Tyr472+) or without (Tyr472−) the C-terminal tyrosine—were identified. Based on the intensities of the corresponding glycopeptides, the majority of mIgA tailpieces are truncated with the ultimate C-terminal Tyr472 missing, whereas tailpieces in circulatory dIgA assemblies preferentially contain this tyrosine. As expected, the Tyr472+/Tyr472– tailpiece sequence variant ratio in the originating serum IgA sample fell between these extremes but is skewed by the high proportion of circulatory IgA represented by monomers. (D) The monomeric IgA tailpiece *N*-glycosylation site exhibited uniform sialylated glycan signatures, while the circulatory dimeric IgA tailpieces exhibited more heterogeneous glycoprofiles, including high-mannose glycan features. (E) Overview of the distinctive features in *N*-glycosylation at Asn459 in Tyr472+ and Tyr472– tailpiece variants across monomeric and J-coupled dimeric molecular forms of circulatory IgA for Donors 5 and 6. Glycan structure categories are represented according to the color scheme shown, with the overall level of sialylation indicated beneath each summary pie chart. The underlying quantitative data for 3D and 3E are given in Supporting Information File 5, providing peak areas of glycopeptides using Skyline.

In both samples of serum-derived mIgA and dIgA for Donors 5 and 6, we again detected the same two tryptic tailpiece-containing peptides referred to as Tyr472+ and Tyr472– (Figure 3C). Although previous studies^37^ have shown that serum-derived IgA contains these two peptides differing by the presence or absence of Tyr472, here we provide evidence that both variants co-occur in both mIgA and dIgA assemblies. However, the relative abundances of the Tyr472+ and Tyr472– tailpiece variants differ substantially between mIgA and dIgA, similar to the difference observed between milk and serum (Figure S3). Specifically, the truncated Tyr472– tailpiece variant was dominant in serum mIgA from Donors 5 and 6, whereas in dIgA, full-length Tyr472+ tailpieces predominated (Figure 3C). Thus, while the ultimate tyrosine residue had been clipped in most of the serum mIgA tailpieces (∼65–75%), Tyr472 was still present at the C-terminus in the majority of the heavy chains present in dIgA assemblies (Figure 3C). The distribution of Tyr472+ and Tyr472– tailpieces in unfractionated serum IgA from both of these donors is also included for comparison in Figure 3C.

Notably, serum mIgA and dIgA showed nearly identical *N*-glycosylation profiles at both CH2 domain sites, Asn263 and Asn337 (Figure S4). *N*-Glycosylation in the tailpiece region (Figure 3), on the other hand, told a different story. As depicted in Figure 3D and summarized in Figure 3E, features of *N*-glycans attached to Asn459 were distinct, especially between dIgA tailpieces in the presence or absence of the C-terminal tyrosine residue. Both Tyr472– and Tyr472+ tailpiece-containing glycopeptides derived from serum mIgA, as well as those from dIgA containing the truncated Tyr472– tailpiece sequence, exhibited similar and relatively simple glycosylation patterns dominated by mature sialylated glycans. In contrast, Tyr472+ tailpiece-containing glycopeptides derived from serum dIgA carried a substantially broader range of *N*-glycan structures (Figure 3D). In particular, high-mannose glycans and hybrid structures were both more abundant, occupancy of Asn459 with hybrid structures increased, and levels of sialylation, fucosylation, and bisecting GlcNAc features were considerably lower among the complex-type glycans (Figure 3E). Although less pronounced, there seems to be also a slightly higher variety in the Tyr472– tailpiece variants of dIgA compared to mIgA.

Interestingly, the serum dIgA Tyr472+ tailpiece Asn459 glycosylation signature shown in Figure 3D qualitatively resembles that observed for the same site in milk SIgA tailpieces (which are effectively all Tyr472+) depicted in Figures 2D and 2E. Specifically, in contrast to other tailpieces that predominantly carried fully processed, sialylated glycans, both serum dIgA (Figures 3D and 3E) and milk SIgA (Figures 2D and 2E) displayed broad Asn459 glycan profiles ranging from high-mannose structures to fully matured complex-type glycans. Both serum dIgA and milk SIgA contain a J-chain disulfide linked to two tailpieces from different IgA subunits via their penultimate Cys471 residues (Figure 1B). By contrast, all mIgA is completely devoid of the J-chain, leaving both tailpiece cysteines presumably free. According to our data, most tailpieces in mIgA assemblies are tyrosine-clipped with the cysteines C-terminally exposed. We cannot exclude the possibility that some mIgA tailpieces are not free, as the extracellular association of IgA with various serum proteins involving disulfides with Cys471 has been reported to occur^39^ and formation of intrasubunit disulfides seems also to be possible,^13^ although we did not find evidence for that here. Together, these observations prompted us to further investigate potential correlated behavior between all three major features of IgA tailpieces. We therefore next aimed to determine whether the IgA tailpieces that are covalently linked to the J-chain—of which there are only two in each polymeric IgA assembly, regardless of oligomer size or biofluid—differ from those which are not bound to the J-chain with respect to *N*-glycan signatures and/or presence of the C-terminal tyrosine.

### Complete Loss of Symmetry in IgA through Coupling with the J-Chain

To investigate potential structural and glycosylation differences between the four tailpieces present in J-coupled dimers of IgA, tryptic digestion was performed under non-reducing conditions to preserve the native redox state of Cys471 residues, keeping disulfide bonds intact. We analyzed glycopeptides generated in this manner from a commercially obtained sample of SIgA purified from pooled human colostrum and from dIgA purified from the serum of Donor 5. Performing the digestion under non-reducing conditions allowed us to distinguish between different tailpiece configurations based on the redox status and disulfide connectivity of Cys471. Within J-coupled polymeric IgA assemblies, Cys471 may either be disulfide-bridged to Cys37 or Cys91 of the J-chain, found as a free cysteine, and/or even disulfide-linked to Cys471 of the adjacent heavy chain from a neighboring IgA subunit^15,18,40,41^ (Figure 4A). All of these tailpiece configurations co-occur in J-coupled dIgA and dimeric SIgA^27^ except for the latter intersubunit Cys471–Cys471 disulfide, which is expected to be present only in the less abundant higher-order polymeric assemblies of IgA. Indeed, all three expected tailpiece configurations—i.e., Cys471­(IgA)–Cys37­(J-chain), Cys471­(IgA)–Cys91­(J-chain), and free Cys471—could clearly be detected in human colostrum SIgA, as supported by the high-quality MS2 fragmentation spectra depicted in Figure S5. In the J-chain-linked tailpieces, the disulfide bridges could be confidently assigned to their respective cysteine pairs. These were easily distinguished from tailpieces featuring free Cys471 residues, as these underwent Cys-alkylation during sample handling.

**Figure 4:**
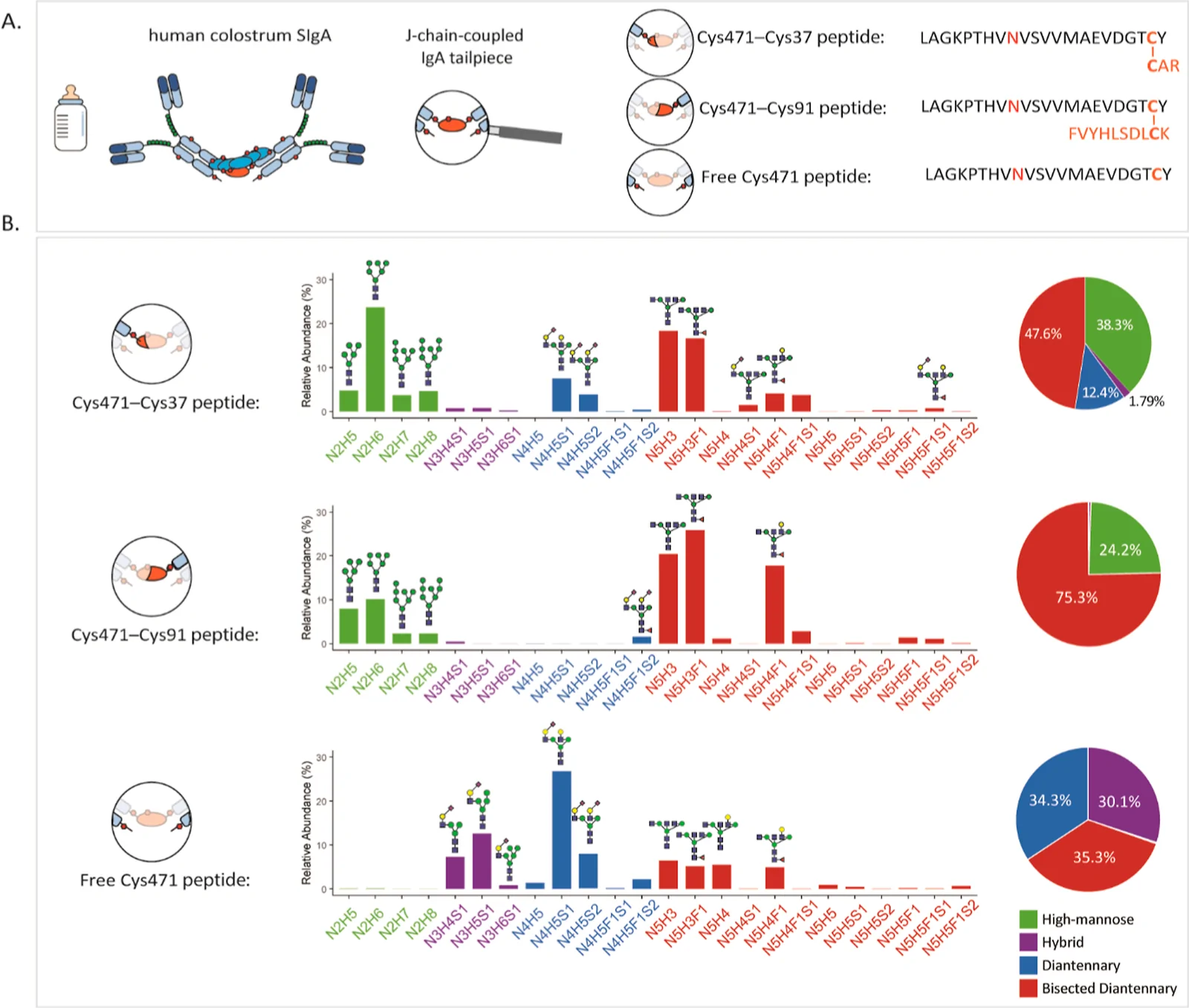
The secretory IgA tailpiece exhibits glycosylation features affected by J-chain coupling. (A) SIgA is predominantly composed of two IgA monomers joined by the J-chain to which pIgR is bound. The IgA tailpiece Cys471 can exist in three distinct states: disulfide-bound to Cys37 of the J-chain, disulfide-bound to Cys91 of the J-chain, or as a free thiol. Tryptic digestion under nonreducing conditions was used to investigate glycopeptides representing each of these states. The resulting distinct SIgA tailpiece-containing glycopeptides detected are depicted. (B) The three states of the tailpiece all carried distinct glycosylation signatures. J-chain-linked peptides (Cys471–Cys37 and Cys471–Cys91 peptides) carried predominantly high-mannose glycans, whereas free tailpieces lacked high-mannose glycans and were enriched in sialylated glycans.

Intriguingly, each SIgA tailpiece configuration exhibited a distinct glycosylation signature (Figures 4B and 5). Tailpieces containing the Cys471–Cys37 IgA–J-chain disulfide bond were relatively enriched in high-mannose and both nonbisected and bisected biantennary glycans. In contrast, SIgA tailpieces linked to the J-chain via the Cys471–Cys91 disulfide bond primarily carried high-mannose and bisected biantennary structures. Notably, high-mannose glycans were abundant exclusively on tailpieces engaged in disulfide linkages to the J-chain, while sialylated glycans were relatively more abundant in unlinked tailpieces in colostrum SIgA (Figure 4B). The SIgA tailpieces containing free Cys471 residues (detected in their alkylated forms) lacked high-mannose glycans and instead harbored hybrid and partially bisected biantennary structures (Figure 4B).

**Figure 5:**
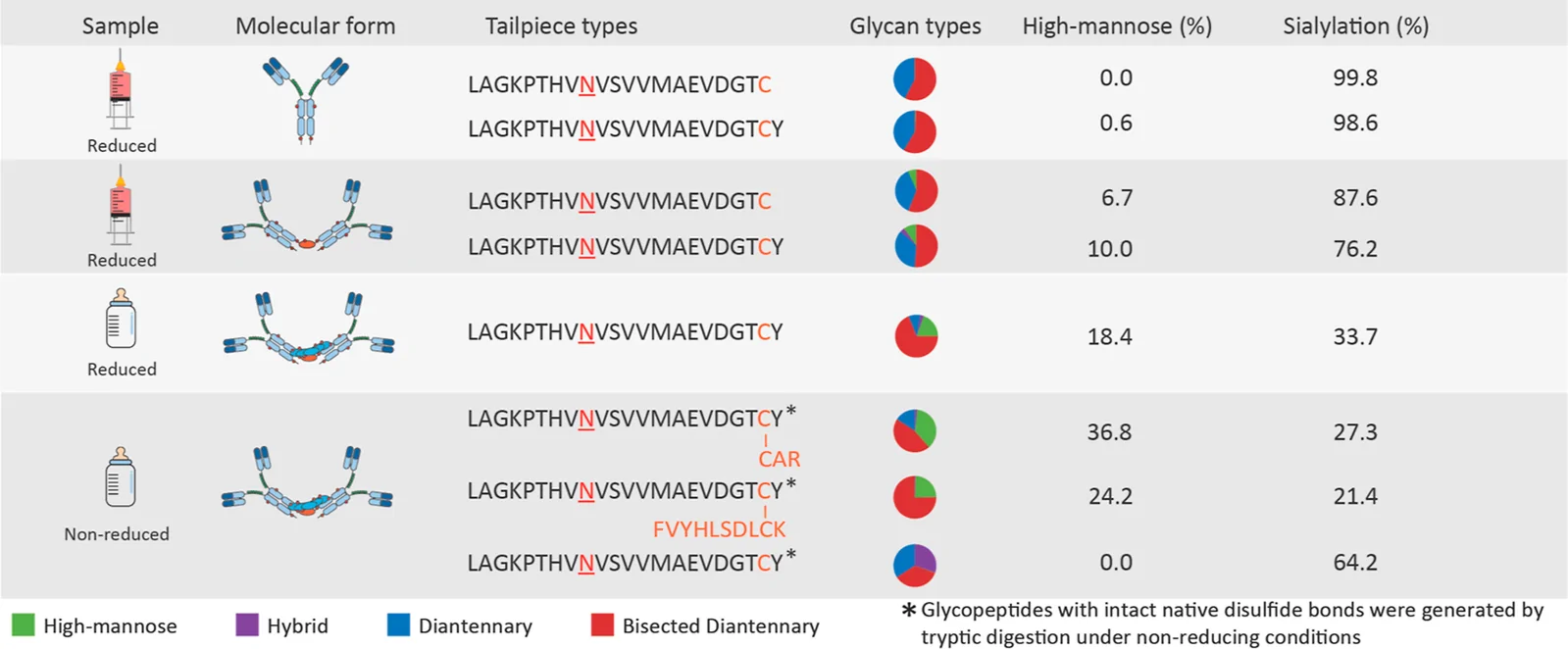
Intricate proteoform heterogeneity in the tailpiece of IgA within and across circulatory and secretory forms. Values represent averages across all measured samples. mIgA and dIgA are shown as the average of Donors 5 and 6. SIgA (reduced) is derived from the average of all measured reduced SIgA samples, while SIgA (nonreduced) is derived from the colostrum SIgA sample. Summary of the findings. IgA exhibits multiple variable features in the tailpiece, including (a) *N*-glycosylation at Asn459; (b) the penultimate Cys471 existing as a free thiol or forming a disulfide bond with either Cys37 or Cys91 of the J-chain; and (c) the presence or absence of the ultimate Tyr472. The occurrence of Tyr472 is distinctive between circulatory and secretory: serum IgA contains both Tyr-positive and Tyr-negative molecular variants, whereas milk IgA exclusively has variants harboring Tyr472. Asn459 glycosylation is highly affected by the state of Cys471: free Cys471 leads to Asn459 consistently harboring mature, sialylated glycans, whereas J-chain-linked Cys471 exhibited a wider variety of glycosylation features, including high-mannose and other more immature glycan structures. Therefore, these glycan signatures seem to highly depend on the microenvironment.

Additionally, we observed that free Cys471 residues were not only chemically alkylated but also exhibited other modifications, such as *S*-sulfinylation (Figure S6A). The diversity of Cys471 modifications, together with the pronounced heterogeneity of Asn459 glycosylation, substantially dispersed the signal of free Cys471 tailpiece-containing glycopeptides. As a result, the overall cumulative detected abundance of alkylated free Cys471 tailpiece peptide species was markedly lower than that of the J-chain-linked Cys471–Cys37 and Cys471–Cys91 disulfide-linked glycopeptides (Figure S6B).

Endogenous levels of circulatory J-coupled dimeric IgA are typically rather low, and samples of dIgA purified from human serum were not commercially available. We therefore utilized the serum of Donor 5 as the most abundant source of serum-derived dIgA (Figure 3B) available to us for further experiments in this comparative analysis of disulfide-linked and free tailpieces. Despite this, the identification of tailpiece-containing glycopeptides in serum-derived dIgA was still hampered by limited sample availability, and consequently our data relied primarily on retention time and MS1 features using the corresponding glycopeptides observed in SIgA as references. The full spectrum of glycan signatures present on each glycopeptide thus may not have been comprehensively captured. Despite this limitation, we were still able to make several informative observations. As shown in Figure S7, both Cys471–Cys37 and Cys471–Cys91 disulfide-linked tailpiece configurations identified above in SIgA were also detected in serum dIgA, with each covalent tailpiece configuration appearing to display a distinct *N*-glycosylation profile. Of the two tailpiece C-terminal sequence variants, only Tyr472+ tailpieces were observed to participate in disulfide bonding with the J-chain (Figure S7). Disulfide-linked Tyr472– tailpieces were not detected in these experiments.

Free Cys471 tailpiece-containing glycopeptides were also not observed from digestion of serum-derived dIgA under nonreducing conditions. We speculate that this may reflect the pronounced molecular heterogeneity of the free tailpiece population, comprising both Tyr472+ and Tyr472– C-terminal sequence variants, multiple Cys471 modification states, and substantial Asn459 glycan heterogeneity. Collectively, this diversity likely diluted the signal of free Cys471-containing tailpiece peptides, rendering them below the detection limit. Taken together, we speculate that Tyr472– tailpieces may be more likely to exist in an unlinked state in J-coupled dimers of IgA present in circulation, although this interpretation currently lacks orthogonal validation. Low overall abundance may also partly account for the lack of detection of disulfide-linked Tyr472– species; however, further experimental confirmation will be needed to substantiate this observation.

Overall, these findings further underscore the extensive structural diversity of IgA and reveal a remarkably broad proteoform landscape. Three features in the IgA tailpiece, namely, *N*-glycosylation signature at site Asn459, Cys471 disulfide connectivity, and the presence/absence of the C-terminal Tyr472, together contribute to the structural heterogeneity of human IgA.

## Discussion

Here, using mass spectrometry-based glycoproteomics, we charted details of the structural heterogeneity occurring within the IgA tailpiece, which are summarized in Figure 5. Although IgA exists in several distinct molecular forms that are differentially distributed across distinct immune compartments, three modifications occurring in the C-terminal tailpiece—namely, (i) the presence or absence of the C-terminal Tyr472 residue, (ii) the redox status of Cys471 (disulfide-linked to either Cys37 or Cys91 of the J-chain, or remaining free), and (iii) the *N*-glycosylation profiles at Asn459—are highly interdependent, often termed PTM cross-talk.

First, when Cys471 is disulfide-bound to Cys37 or Cys91 of the J-chain, the glycans attached to Asn459 exhibit broader structural heterogeneity, including high-mannose, hybrid, and mature sialylated biantennary glycans with and without bisection. In contrast, when Cys471 remains free, the attached tailpiece *N*-glycans tend to be fully processed complex-type structures enriched in sialylation and with almost no high-mannose glycans. On the basis of known pathways of immunoglobulin biosynthesis and protein *N*-glycosylation in humans,^42^ we hypothesize that the formation of disulfide bonds between the IgA heavy chain and the J-chain in the endoplasmic reticulum may sterically restrict subsequent glycan maturation within the Golgi apparatus, leading to retention of high-mannose structures. Conversely, IgA tailpieces not involved in disulfide linkages would have greater flexibility and solvent exposure, allowing for complete glycan processing including sialylation, thereby yielding fully matured complex glycans. This hypothesis is directly supported by the serum mIgA tailpiece glycosylation profiles (Figure 3D). Additionally, we found mostly complex sialylated glycans and no high-mannose structures attached to Asn71 of the J-chain, consistent with the lack of protein contacts and steric hindrance at this site as also revealed by the cryo-EM structures of SIgA reported by Kumar et al.^17,18^

Second, although serum dIgA and milk SIgA both contain two IgA tailpieces disulfide-linked to the J-chain, they exhibit different glycan signatures across *N*-glycosylation sites in the Fc domain. Notably, their CH2 domain glycosylation profiles show almost no similarity, as revealed by comparison between Figures 2D and S4. In contrast, differences in tailpiece Asn459 *N*-glycosylation between SIgA (Figure 2D) and serum dIgA Tyr472+ tailpieces (Figure 3D) are more nuanced, with a broad range of *N*-glycan structures from high-mannose to complex-type found on both. The individual glycans dominating this site were still rather distinct between SIgA and serum-derived dIgA tailpieces, with sialic acid capping nearly every structure in serum but largely absent from milk SIgA tailpiece glycans. Biantennary structures without bisection were notably higher in abundance relative to those with bisecting GlcNAc in serum dIgA as compared to milk SIgA (Figures 2E and 3E). Even larger discrepancies emerge between serum dIgA and milk SIgA tailpiece Asn459 glycosylation when the glycan signatures of Tyr472– tailpieces present in serum dIgA are considered (Figure 3D). A possible difference in the plasma half-life of dimeric versus monomeric IgA may partly explain the observed distinct glycosylation of these assemblies. It has been demonstrated that in some organisms, dimeric IgA is quickly removed from plasma to the bile by the pIgR expressed in hepatocytes. In humans, this pathway seems also active in transporting dIgA, thereby enriching SIgA in human bile, albeit to a lesser degree than in rodents.^43–45^ Still, the catabolic rates might be different, e.g., through removal in the liver mediated by the asialoglycoprotein receptor. Regardless, there are yet no indications that IgA glycosylation is altered by other cells than B plasma cells. Taken together, these differences in *N*-glycosylation indicate that at least some of the J-coupled dimeric IgA assemblies which end up in circulation and those that become SIgA after pIgR-mediated transport into human milk may originate from distinct populations of J + IgA-secreting cells, possibly through different glycoenzyme activities or more likely due to differential substrate accessibility for these enzymes.^26^

By contrast, serum-derived mIgA and dIgA exhibited highly similar glycan profiles (Figure 3D,E), with one notable difference: in dIgA, full-length tailpieces retaining the C-terminal Tyr472 residue carried relatively immature glycans characterized by an elevated abundance of high-mannose glycans and lower sialylation. In contrast, all other serum IgA tailpieces (i.e., truncated Tyr472–dIgA tailpieces and both mIgA tailpiece variants) mainly contained mature, sialylated glycans. These observations indicate that the presence of the J-chain and its disulfide bridge with the IgA heavy chain likely alters the local environment of the tailpiece, thereby affecting glycan maturation at Asn459. The similarities observed between serum mIgA and dIgA glycosylation signatures suggest at least some fraction of these two circulatory IgA assemblies in humans could originate from the same population of J + IgA-secreting cells. This is consistent with recent results from MS-based profiling of assembly-specific circulatory IgA1 clonal repertoires demonstrating shared clonality between mIgA1 and dIgA1, hinting at their common cellular origins.^4^ The IgA tailpiece glycan signature and assembly outcome could depend on the involvement of protein disulfide isomerases and/or other molecular chaperones (e.g., MZB1, BiP, Erp44), given that the assembly and secretion of J-chain-coupled polymeric IgA complexes is known to be post-translationally controlled.^46,47^ For example, Xiong et al. showed that the absence of MZB1 reduced secretion of J-chain-coupled dimeric IgA concomitant with increased amounts of monomeric IgA, although these data were only obtained *in vitro* and in mice.^46^ However, the complete mechanistic picture including the proteins involved in regulating mIgA versus dIgA assembly and secretion has yet to emerge.^48,49^

Finally, we observed that milk SIgA almost exclusively contained full-length tailpieces with Tyr472 present (Figure S3), regardless of whether Cys471 was disulfide-linked to the J-chain or not (Figure 4B). Serum, however, contained heterogeneous populations of mIgA and dIgA each with tailpieces that retained the C-terminal tyrosine as well as truncated Tyr472– variants (Figure 3). The presence or absence of Tyr472 appears to be strongly associated with the fluid environment in which IgA resides. Therefore, it is tempting to propose that this clipping most likely arises from postsecretory enzymatic processing of IgA by a carboxypeptidase present and/or active only in serum^29^ and not at mucosal sites. Consequently, all IgA heavy chains are initially synthesized with a C-terminal tyrosine residue, with the Cys471–Tyr472 peptide bond of a subset of tailpieces in both mIgA and dIgA assemblies secreted into circulation subsequently hydrolyzed. C-terminal tyrosine clipping likely occurs preferentially on solvent-accessible, unbound IgA tailpieces, as those covalently linked to the J-chain would be sterically protected from enzymatic access. In future studies, it would be interesting to explore which peptidase is responsible for this clipping, and to dissect what the functional consequences are of the loss of this residue. In nature, a well-known example of a specific tyrosine-cleaving enzyme is the Vasohibin/SVBP complex that specifically cleaves the terminal tyrosine from α-tubulin, producing detyrosinated tubulin,^50^ but more likely in serum it could be one of the less specific chymotrypsin-like serine proteases with a preference for aromatic sites that is responsible for this cleavage.

## Conclusion

In conclusion, we here provide a comprehensive overview of the heterogeneous glycoproteoform landscape of the human IgA tailpiece (Figure 5), which strongly depends on its compartmental localization as well as on its molecular form. We dissected variations in Asn459 *N*-glycosylation, redox status and disulfide connectivity to the J-chain of the penultimate Cys471, and presence or absence of the ultimate C-terminal Tyr472 residue. Our results demonstrate that these structural variations are intrinsically interconnected and that the combined signatures of these three tailpiece features define distinct compartment-specific molecular forms of IgA. The structural analysis presented here may also offer valuable insights needed for the development of IgA-based therapeutic antibodies with unique applications in mucosal vaccine development and intervention strategies for infectious and immune-related diseases.^51–53^

## Experimental Section

### Chemicals, Materials, and Samples

Chloroacetamide (CAA), iodoacetamide (IAA), tris­(2-carboxyethyl) phosphine hydrochloride (TCEP), Tris, trypsin, Lys-C, sodium deoxycholate (SDC), and sodium acetate were obtained from Sigma-Aldrich (Steinheim, DE). Milli-Q water was generated from a Merck Milli-Q IQ 7003 system (Darmstadt, DE). The Oasis PRiME HLB plate was purchased from Waters (Etten-Leur, NL). Acetonitrile and 0.1% formic acid (FA) were obtained from Biosolve (Valkenswaard, NL). Trifluoroacetic acid (TFA) was obtained from Honeywell International Inc. (Charlotte, NC, USA).

The matched human serum and milk samples of Donors 1–4 used in this study are a subset of the healthy control group from a larger longitudinal follow-up to the COVID MILK study.^36,54^ This study was approved by the Medical Ethics Committee of Amsterdam UMC, and written informed consent was obtained from all participants before participation in either the original or follow-up COVID MILK studies. The individual serum sample of Donor 5 was provided by Sanquin (Amsterdam, NL) and was collected with informed consent from the anonymous healthy volunteer in accordance with ethical approval from the Sanquin Ethical Advisory Board. The individual K_2_EDTAplasma sample of Donor 6 was obtained commercially from Zen-Bio (Durham, NC, USA) and was collected from a healthy male adult undergoing an elective procedure. Purified IgA from pooled normal human colostrum was obtained from Merck (Darmstadt, DE) as a lyophilized powder and was reconstituted according to the manufacturer’s recommendations. All individual biological samples were stored at −80 °C until further analysis.

### IgA Affinity Purification

IgA was purified from all individual human milk and serum/plasma samples using CaptureSelect IgA Affinity Matrix (Thermo Fisher Scientific), which binds all human IgA subclasses, allotypes, and molecular forms. For each sample, a volume of affinity resin slurry (1:1 beads/buffer) appropriate to efficiently capture an expected IgA quantity of 40 μg (i.e., with a binding capacity 4-fold higher) was prepared. The bead slurry was transferred into a Pierce spin column with a screw cap and equilibrated by repeated wash cycles (minimum of three), with addition of 150–200 μL of phosphate-buffered saline (PBS) followed by centrifugation at 500 × *g* for 1 min. The columns were then plugged prior to addition of the sample.

For each sample, 20 μL of human serum or 50 μL of human milk (both equivalent to the expected IgA quantity of 40 μg) was added to the prepared affinity resin together with 150 μL of PBS. Samples were incubated at room temperature for 1 h while shaking at 750 rpm in a thermomixer to allow all IgA to be affinity-captured. After incubation, the plugs were removed from the spin columns, and the initial flow-through containing unbound proteins was collected by centrifugation (1 min, 500 × *g*). The beads were then washed twice with PBS followed by two additional washes with Milli-Q water to fully remove all unbound (i.e., non-IgA) proteins and any residual salts. The affinity-captured IgA from each sample was eluted from the affinity matrix in the plugged spin columns by adding 100 μL of 0.4% (v/v) formic acid and incubating at room temperature for 10 min with shaking at 750 rpm and subsequently collected by centrifugation at 1000 × *g* for 1 min. The eluate was immediately neutralized by addition of 20 μL of 1 M Tris–HCl, pH 8.0. Purified IgA was stored briefly at 4 °C before either being analyzed by single-molecule mass photometry (MP), used directly for proteolytic digestion, or subjected to subsequent size-exclusion chromatography (SEC) fractionation prior to mass spectrometric analysis.

### Mass Photometry of Intact IgA Assemblies Affinity-Purified from Serum

The molecular composition and assembly distribution of circulatory IgA affinity-purified from the serum/plasma of Donors 5 and 6 were determined using single-molecule mass photometry (MP), a light scattering-based technique that accurately measures the mass of single molecules in a sample. Particles are detected upon landing on the glass coverslip surface, with the ratiometric contrast value (proportional to mass) recorded for each such “event”. All detected particles from a single measurement can be plotted as a mass histogram, enabling quantitative comparison between co-occurring molecular assembly populations. All MP experiments were performed on a Refeyn OneMP mass photometer (Refeyn Ltd., Oxford, UK).

Borosilicate glass microscope coverslips (24 × 50 mm) were cleaned by sonication in two sequential iterative rounds with isopropanol followed by Milli-Q water. Silicone cell culture gaskets cut into sets of six wells (1 mm deep × 3 mm in diameter) were placed in the center of the clean, dry coverslips. Prior to each sample measurement, 12 μL of PBS was placed in a coverslip well to refocus the instrument. Then 3 μL of sample was added to the PBS in the well by pipetting up and down, with a final concentration of ∼10–100 nM in the droplet. Measurements were recorded for 60 s in the AcquireMP software (Refeyn) using medium field-of-view settings. Calibration was performed using an in-house mixture of four proteins with accurate masses previously determined by mass spectrometry: IgG half-body (IgG4Δhinge-L368A), 73 kDa; IgG1-Campath, 149 kDa; apoferritin, 513 kDa; and IgM, 1006 kDa.

Data were calibrated, refined, and processed in the DiscoverMP software (Refeyn) and then exported for further analysis and plotting using an in-house Python library. As shown in Figure 3B, mass histogram peaks were fit with Gaussian distributions to determine the mass and total number of detected molecules in each monomeric or J-coupled dimeric IgA assembly population. Molecule counts were adjusted considering each J-coupled dimer contains two IgA subunits (i.e., twice as much IgA heavy chain as a monomer),^4,5^ with the resulting fractions of total circulatory IgA present as monomer or J-coupled dimer for both Donors 5 and 6 indicated in the bar charts shown in Figure 3B.

### SEC Separation of IgA from Human Serum Samples

Size exclusion chromatography (SEC) was used to separate intact monomeric and J-coupled dimeric IgA assemblies affinity-purified from the serum/plasma of Donors 5 and 6. The SEC method and conditions used were optimized and validated previously for the separation of mIgA and dIgA by mass photometry and bottom-up LC–MS/MS proteomics analysis.^4^ A Superdex 200 Increase 10/300 GL column (30 cm length × 10 mm i.d.; 8.6 μm particle size; Cytiva, Marlborough, Massachusetts, USA) was used for all SEC separations. This column has a fractionation range of 10–600 kDa with optimized resolution over the typical antibody monomer/dimer molecular weight range of 100–300 kDa, making it ideal for separation of mIgA and dIgA. To maximize the quantity of purified serum dIgA available for downstream glycoproteomics analysis, affinity-purified intact IgA samples for Donors 5 and 6 were prepared for SEC using double the amount of serum/plasma as normal for the same elution conditions and volumes (see the section “IgA Affinity Purification” above). The resulting concentrated eluate containing intact circulatory IgA of each donor was pH-adjusted as normal with a final volume of 120 μL, of which 100 μL was injected for SEC.

All SEC experiments were performed using an Agilent 1260 Infinity HPLC system (Agilent Technologies, Waldbronn, DE) equipped with an autosampler, binary pump, column oven, multiwavelength detector, and fraction collector. The autosampler and fraction collector were both maintained at 4 °C, while the column temperature was set to 17 °C. Proteins were eluted isocratically within 120 min with PBS supplemented with 500 mM NaCl at a flow rate of 250 μL/min. Absorbance at 280 nm was recorded throughout each SEC run. Fractions were collected every 1 min (250 μL per fraction) between 30 and 50 min, yielding 20 fractions per run.

IgA molecular forms were assigned to the two major peaks in each SEC profile (data not shown) according to the elution time ranges previously established^4^ and supported by MP and proteomics analysis results. Matching previous size exclusion chromatography results,^4^ the SEC peak corresponding to J-chain-coupled dimers of IgA (∼340 kDa) eluted predominantly over 39–40 min, while maximal elution of monomeric IgA (∼160 kDa) occurred in the 45–46 min window. These elution windows were used to define dIgA and mIgA SEC fractions for downstream glycoproteomic and tailpiece analyses.

### IgA Digestion

For a standard digestion, IgAs were diluted in digestion buffer containing 100 mM Tris–HCl (pH 8.5), 1% w/v sodium deoxycholate (SDC), 5 mM TCEP, and 30 mM CAA. Lys-C was then added to digest plasma or proteins for 3 h at an enzyme-to-protein ratio of 1:75 (w/w) at 37 °C, and the resulting peptide mixtures were further digested overnight at 37 °C by trypsin (1:20, w/w). The next day, SDC was removed via acid precipitation (0.5% trifluoroacetic acid) (TFA). The peptides were desalted using an Oasis PRiME HLB plate, then dried, and stored at −80 °C before MS analysis.

For a nonreduced digestion, the procedure was adapted from previously reported protocols. IgA samples were mixed with 8 M urea in 50 mM ammonium bicarbonate, 8 M guanidine hydrochloride, and iodoacetamide to reach a final protein concentration of 2 mg/mL, a final iodoacetamide concentration of 2.5 mM, and a final guanidine-HCl concentration of 1 M. Samples were incubated at 50 °C for 30 min. A 20 μL aliquot of each sample was digested with trypsin/Lys-C at an enzyme-to-substrate ratio of 1:20 (w/w) at 37 °C for 30 min. The samples were then diluted 4-fold with 50 mM ABC and further digested with additional trypsin/Lys-C at the same enzyme-to-substrate ratio for 1 h at 37 °C. Digestion was stopped by adding formic acid to a final concentration of 1% (v/v) prior to LC–MS analysis.

### HILIC-LC-MS/MS Method

Shotgun HILIC–LC–MS/MS was performed by means of an UltiMate 3000 UHPLC system (Thermo Fisher Scientific) coupled to an Orbitrap Eclipse or an Orbitrap Exploris 480 mass spectrometer (Thermo Fisher Scientific).^35^ A total of 600 ng of protein digests were loaded onto a trap column (PepMap Neo Trap Cartridge, 174500, 300 μm inner diameter, 5 mm; Thermo Fisher Scientific) coupled to a 75 μm inner diameter × 25 cm analytical column (in-house packed with HALO penta-HILIC, 2.7 μm, Advanced Materials Technology, USA). The mobile-phase solvent A consisted of 0.1% formic acid (FA) and 0.01% DFA in water, and solvent B consisted of 0.1% FA and 0.01% DFA in acetonitrile (ACN). Trapping was performed at a flow rate of 30 μL/min for 1 min with 0% B, and peptides were eluted using a flow of 300 nL/min for 130 min with the following gradient: 0–1 min: 95–95% B, 1–8 min: 95–82% B, 8–85 min: 82–64% B, 85–150 min: 64–40% B, 150–155 min: 18% B, 155–180 min: hold at 95% B. For MS settings, the source voltage was set at 2000 V, and the ion transfer tube temperature was maintained at 275 °C. The initial scan parameters were established with a scan range of *m*/*z* 350–2000 and a resolution of 120,000, utilizing automatic gain control (AGC) set to standard with a maximum injection time of 50 ms. Ions with an intensity over 5 × 10^3^, with charge states from 2+ to 8+, were selected for HCD fragmentation with a dynamic exclusion time of 6 s. For HCD fragmentation, MS/MS acquisition was conducted in the HCD cell, with readings captured on the Orbitrap mass analyzer. This setup provided a resolution of 60,000 for *m*/*z* 120–4000, AGC set to standard, maintaining a maximum injection time of 50 ms and a normalized collision energy of 15%. Additionally, a target mass list was utilized, representing the IgA glycopeptides analyzed in this study, as detailed in Supporting Information File 1.

### Data Analysis

Raw files obtained from the LC–MS/MS experiments were processed using Byonic (version 4.3.4, Protein Metrics Inc.) and searched against the amino acid sequences of human IgA, J-chain, and polymeric immunoglobulin receptor (Supporting Information Data File 2).

For standard digestion conditions (full reduction), the search parameters were set as follows: a specific tryptic digest with a maximum of three missed cleavages; a fixed modification of cysteine residues (+57.021 Da) and variable modifications including methionine oxidation (+15.995 Da), phosphorylation (+79.9663 Da), conversion of glutamine to pyroglutamate (−17.0265 Da), and conversion of glutamic acid to pyroglutamate (−18.0106 Da); variable glycosylation was used for the database search; and an error tolerance of 10 ppm in MS and 20 ppm in MS/MS.

For digestion under nonreducing conditions, the Byonic search included two steps. In step 1, peptides with free Cys were searched with the following parameters: a specific tryptic digest with a maximum of four missed cleavages; Disulfide and Trisulfide search options were not enabled; no fixed modification was set, and variable modifications included carbamidomethylation of cysteine (+57.0215 Da), phosphorylation (+79.9663 Da, on S, T, Y), oxidation of methionine/tryptophan (+15.9949 Da, on M, W), conversion of glutamine to pyroglutamate (−17.0265 Da), conversion of glutamic acid to pyroglutamate (−18.0106 Da), acetylation of cysteine (+42.0106 Da), glutathionylation of cysteine (+305.0682 Da), S-nitrosylation of cysteine (+28.990 Da), sulfinylation of cysteine (+31.9898 Da), sulfonylation of cysteine (+47.9847 Da), and oxidation of cysteine (+15.9949 Da); variable glycosylation was used for the database search, and the error tolerance was set at 10 ppm for MS and 20 ppm for MS/MS.

In step 2, peptides linked via disulfide bonds were searched with the following parameters: a specific tryptic digest with a maximum of three missed cleavages; the Disulfide search option was enabled (disulfide protein groups set to 1, 2), while Trisulfide, DSS cross-link, and custom cross-link search options were not enabled; variable modifications included oxidation of methionine/tryptophan (+15.9949 Da, on M, W), deamidation of asparagine (+0.984016 Da, on N), and conversion of glutamine to pyroglutamate (−17.0265 Da, on protein N-terminal Q); the precursor mass tolerance was set at 10 ppm, and the fragment mass tolerance (qtof_hcd mode) at 20 ppm, with the protein-level FDR cutoff set at 1%.

For the glycopeptides obtained via Byonic, the glycopeptides carrying certain glycan structures and their retention times were manually validated by using Freestyle.

Complete Byonic search results for the colostrum-derived sample, achieving an overall (glyco- and nonglycopeptide) sequence coverage of 76.5% for the IgA heavy chain, are provided as a representative example in Supporting Information File 3. Representative nonglycopeptide identification MS2 spectra are provided in Supporting Information File 4. The raw files obtained from the LC–MS/MS experiments were processed using Byonic (version 4.3.4, Protein Metrics Inc.) to search against the amino acid sequences of human IgA, J-chain, and polymeric immunoglobulin receptor (Supporting Information File 2). The search parameters were set as follows: (1) a specific tryptic digest with a maximum of three missed cleavages; (2) a fixed modification of cysteine residues (+57.021 Da) and variable modifications including methionine oxidation (+15.995 Da), phosphorylation (+79.9663 Da), conversion of glutamine to pyroglutamate (−17.0265 Da), and conversion of glutamic acid to pyroglutamate (−18.0106 Da); (3) variable glycosylation used for the database search; (4) an error tolerance of 10 ppm in MS and 20 ppm in MS/MS. Glycan structural assignments, including glycan branch isomers, were inferred from the combined evidence of characteristic chromatographic retention times and structure-informative MS^2^ fragmentation patterns, including diagnostic fragment ions, and were manually validated using Freestyle.

Skyline (v3.7.0.11317) was used to perform relative quantification. The integrations acquired in this manner were reviewed to meet the following standards: (1) an error of ≤5 ppm relative to the theoretical mass; (2) exhibiting an idotp value ≥0.80 when compared with the theoretical isotopic pattern; (3) elution time falling within a range of ±2 min around the average retention time for the corresponding peptide; and (4) no observable instances of overlapping isotopic peaks. Hence, only glycopeptides accurately identified in Freestyle were permitted for quantification using Skyline. The determination of chromatographic peaks is needed to strictly adhere to retention time regulations. For baseline-separated individual peaks with a width of 30 s, the peak area was determined within this time frame. For peaks that did not achieve baseline separation, the peak area was defined by the valley of the overlapping peaks, extending approximately 30 s forward or backward from the demarcation point. Therefore, the quantification of each glycopeptide was achieved by the peak areas of the corresponding glycopeptide MS spectra (Supporting Information File 5).

Relative abundances were calculated based on the summed peak areas of the detected glycopeptides. For each sample, all glycopeptide peak areas were summed to obtain the total signal, and the contribution of each glycan or glycopeptide was expressed as a percentage of the total. For summary pie charts, glycopeptides were grouped according to glycan structural categories (e.g., high-mannose, hybrid, and nonbisected and bisected biantennary complex-type). The summed peak areas within each category were then expressed as percentages of the total signal. Sialylation (%) was calculated as the proportion of the total glycopeptide signal derived from glycans containing at least one sialic acid residue. All glycopeptides with one or more sialic acids were included in this calculation regardless of the number of sialic acid residues present.

## Supplementary Material













## Data Availability

Data associated with the manuscript have been deposited in the MassIVE repository with the data set identifier MSV000100159 [10.25345/C5ZS2KS7M] and can additionally be downloaded via file transfer protocol with the following link: ftp://massive-ftp.ucsd.edu/v11/MSV000100159/. The unpublished data set is available to reviewers by logging in to the MassIVE repository with the username “MSV000100159” and password “a”.

## Notes

Mass and charge ranges of IgA glycopeptides used in the MS/MS analysis (XLSX)
Used FASTA files (PDF)
Peak areas of glycopeptides obtained using Skyline (XLSX)
Overview of non-modified peptide identifications (PDF)
Peak areas of glycopeptides obtained using Skyline (XLSX)
Additional experimental details, data, and methods including glycoproteomics data and data analysis (PDF)
