# Supplementary material for: Intricate Proteoform Heterogeneity in the Tailpiece of IgA within and across Its Circulatory and Secretory Configurations
Source: J Am Chem Soc. 2026 Oct 21;148(43):46733–46. doi: 10.1021/jacs.6c12113 (PMC13741831; doi:10.1021/jacs.6c12113)
Supplement: Supplementary file 1 [file ja6c12113_si_002.pdf]

```
>P01876-1|IGHA1_HUMAN
ASPTSPKVFPLSLCSTQPDGNVVIACLVQGFFPQEPLSVTWSESGQGVTARNFPPSQDAS
GDLYTTSSQLTLPATQCLAGKSVTCHVKHYTNPSQDVTVPCPVPSTPPTPSPSTPPTPSP
SCCHPRLSLHRPALEDLLLGSEANLTCTLTGLRDASGVTFTWTPSSGKSAVQGPPERDLC
GCYSVSSVLPGCAEPWNHGKTFTCTAAYPESKTPLTATLSKSGNTFRPEVHLLPPPSEEL
ALNELVTLTCLARGFSPKDVLVRWLQGSQELPREKYLTWASRQEPSQGTTTFAVTSILRV
AAEDWKKGDTFSCMVGHEALPLAFTQKTIDRLAGKPTHVNVSVVMAEVDGTCY

>IGHA2M1
ASPTSPKVFPLSLDSTPQDGNVVVACLVQGFFPQEPLSVTWSESGQNVTARNFPPSQDAS
GDLYTTSSQLTLPATQCPDGKSVTCHVKHYTNPSQDVTVPCPVPPPPPCCHPRLSLHRPA
LEDLLLGSEANLTCTLTGLRDASGATFTWTPSSGKSAVQGPPERDLCGCYSVSSVLPGCA
QPWNHGETFTCTAAHPELKTPLTANITKSGNTFRPEVHLLPPPSEELALNELVTLTCLAR
GFSPKDVLVRWLQGSQELPREKYLTWASRQEPSQGTTTFAVTSILRVAAEDWKKGDTFSC
MVGHEALPLAFTQKTIDRLAGKPTHVNVSVVMAEVDGTCY

>IGHA2M2
ASPTSPKVFPLSLDSTPQDGNVVVACLVQGFFPQEPLSVTWSESGQNVTARNFPPSQDAS
GDLYTTSSQLTLPATQCPDGKSVTCHVKHYTNSSQDVTVPCRVPPPPPCCHPRLSLHRPA
LEDLLLGSEANLTCTLTGLRDASGATFTWTPSSGKSAVQGPPERDLCGCYSVSSVLPGCA
QPWNHGETFTCTAAHPELKTPLTANITKSGNTFRPEVHLLPPPSEELALNELVTLTCLAR
GFSPKDVLVRWLQGSQELPREKYLTWASRQEPSQGTTTYAVTSILRVAAEDWKKGETFSC
MVGHEALPLAFTQKTIDRLAGKPTHINVSVVMAEADGTCY

>sp|P01833|PIGR_HUMAN Polymeric immunoglobulin receptor OS=Homo sapiens
OX=9606 GN=PIGR PE=1 SV=4
MLLFVLTCLLAVFPAISTKSPIFGPEEVNSVEGNSVSITCYYPPTSVNRHTRKYWCRQGA
RGGCITLISSEGYVSSKYAGRANLTNFPENGTFVVNIAQLSQDDSGRYKCGLGINSRGLS
FDVSLEVSQGPGLLNDTKVYTVDLGRTVTINCPFKTENAQKRKSLYKQIGLYPVLVIDSS
GYVNPNYTGRIRLDIQGTGQLLFSVVINQLRLSDAGQYLCQAGDDSNSNKKNADLQVLKP
EPELVYEDLRGSVTFHCALGPEVANVAKFLCRQSSGENCDVVVNTLGKRAPAFEGRILLN
PQDKDGSFSVVITGLRKEDAGRYLCGAHSDGQLQEGSPIQAWQLFVNEESTIPRSPTVVK
GVAGGSVAVLCPYNRKESKSIKYWCLWEGAQNGRCPLLVDSEGWVKAQYEGRLSLLEEPG
NGTFTVILNQLTSRDAGFYWCLTNGDTLWRTTVEIKIIEGEPNLKVPGNVTAVLGETLKV
PCHFPCKFSSYEKYWCKWNNTGCQALPSQDEGPSKAFVNCDENSRLVSLTLNLVTRADEG
WYWCGVKQGHFYGETAAVYVAVEERKAAGSRDVSLAKADAAPDEKVLDSGFREIENKAIQ
DPRLFAEEKAVADTRDQADGSRASVDSGSSEEQGGSSRALVSTLVPLGLVLAVGAVAVGV
ARARHRKNVDRVSIRSYRTDISMSDFENSREFGANDNMGASSITQETSLGGKEEFVATTE
STTETKEPKKAKRSSKEEAEMAYKDFLLQSSTVAAEAQDGPQEA

>sp|P01591|IGJ_HUMAN Immunoglobulin J chain OS=Homo sapiens OX=9606
GN=JCHAIN PE=1 SV=4
MKNHLLFWGVLAVFIKAVHVKAQEDERIVLVDNKCKCARITSRIIRSSEDPNEDIVERNI
RIIVPLNNRENISDPTSPLRTRFVYHLSDLCKKCDPTEVELDNQIVTATQSNICDEDSAT
ETCYTYDRNKCYTAVVPLVYGGETKMVETALTPDACYPD
```
