# Supplementary material for: Intricate Proteoform Heterogeneity in the Tailpiece of IgA within and across Its Circulatory and Secretory Configurations
Source: J Am Chem Soc. 2026 Oct 21;148(43):46733–46. doi: 10.1021/jacs.6c12113 (PMC13741831; doi:10.1021/jacs.6c12113)

Colostrum IgA

Overview of non-modified peptide identifications

Non-glycosylated peptides derived from colostrum IgA could also be effectively retained and identified under the HILIC-LC-MS/MS conditions used in this study. The non-glycopeptides eluted across a retention time range of approximately 9.6–19.9 min, with high-quality MS2 spectra and well-resolved b/y ion series. Several representative MS2 spectra of typical non-glycosylated peptides are shown below.

Sequence = NFPPSQDASGDLYTTSSQLTLPATQC[+57]LAGK, z=3, retention time=15.9212

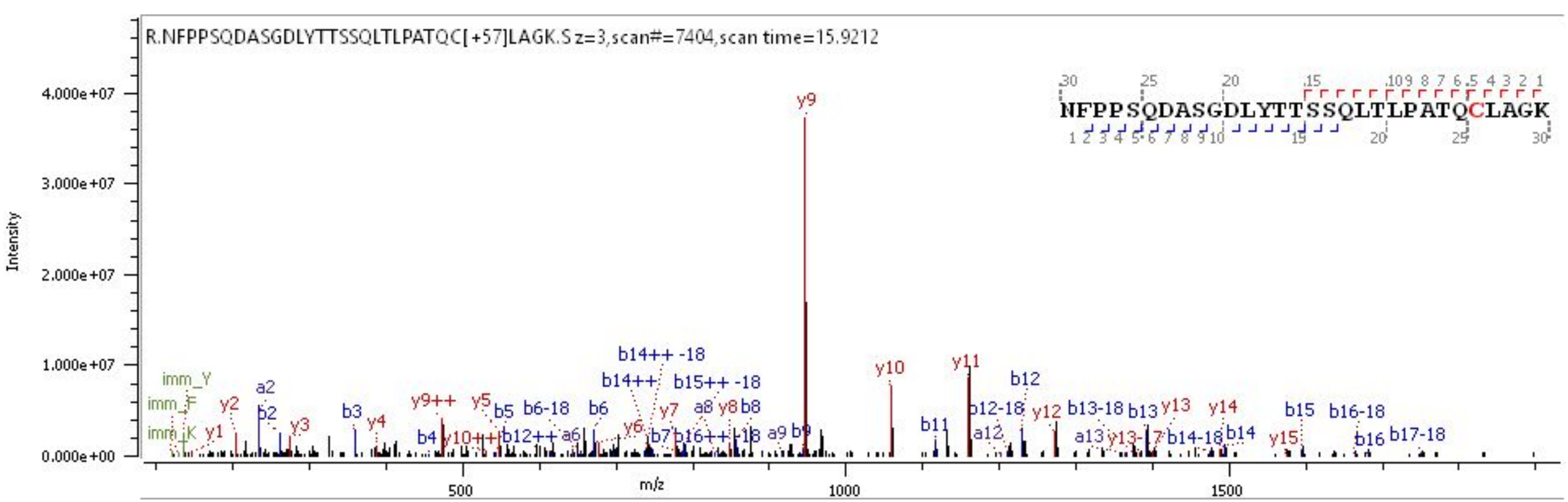

Sequence = DASGVTFTWTPSSGKSAVQGPPER, z=3, retention time=15.7766

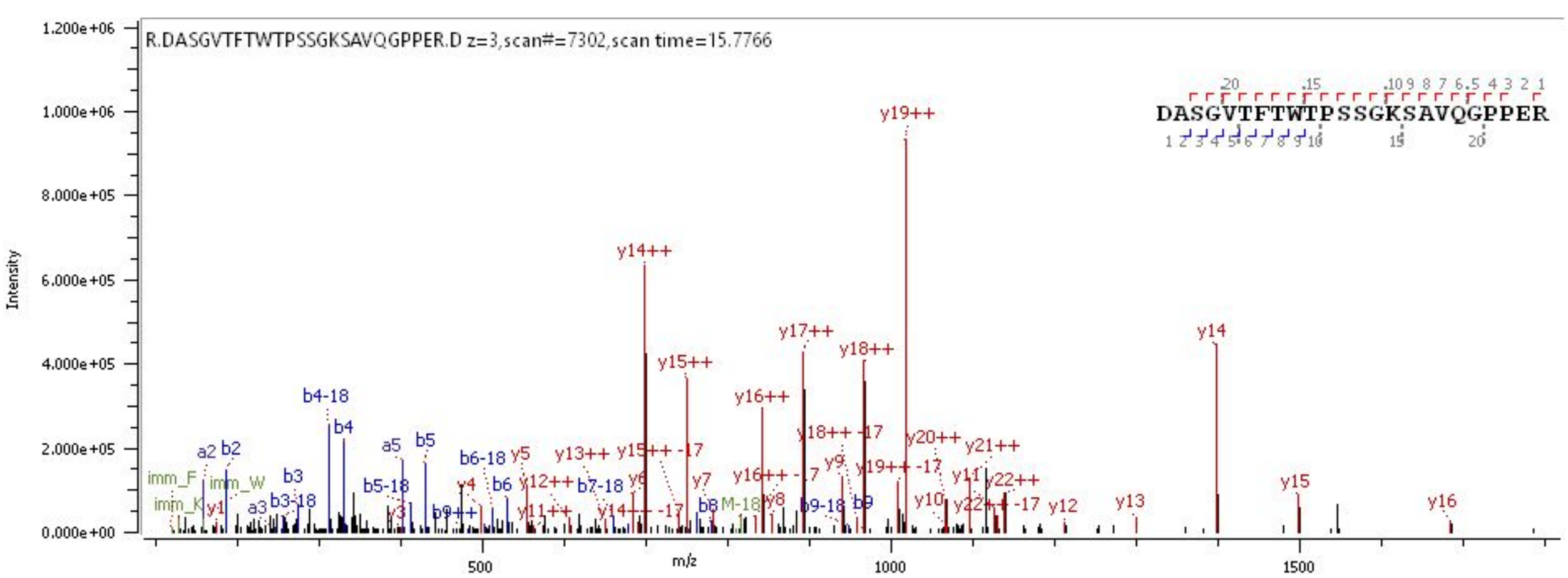

Sequence = DLC[+57]GC[+57]YSVSSVLPGC[+57]AEPWNHGK, z=3, retention time=15.0299

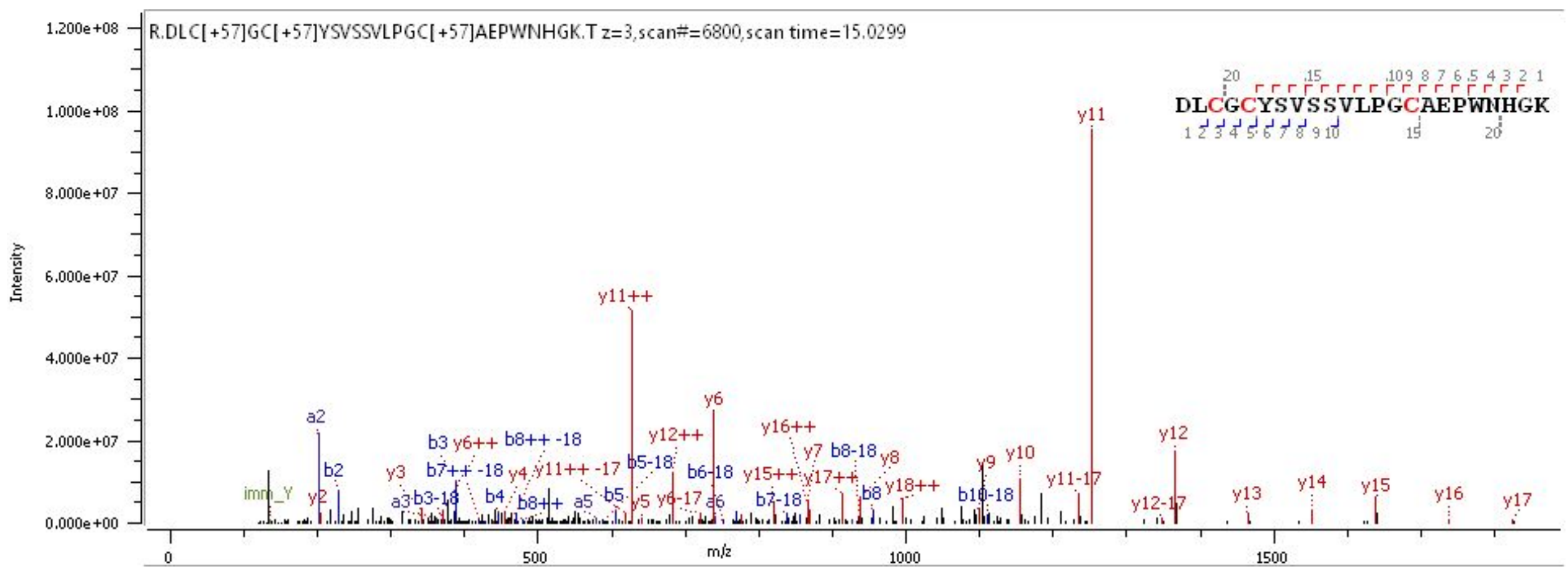

Sequence = TPLTATLSK, z=2, retention time=9.5752

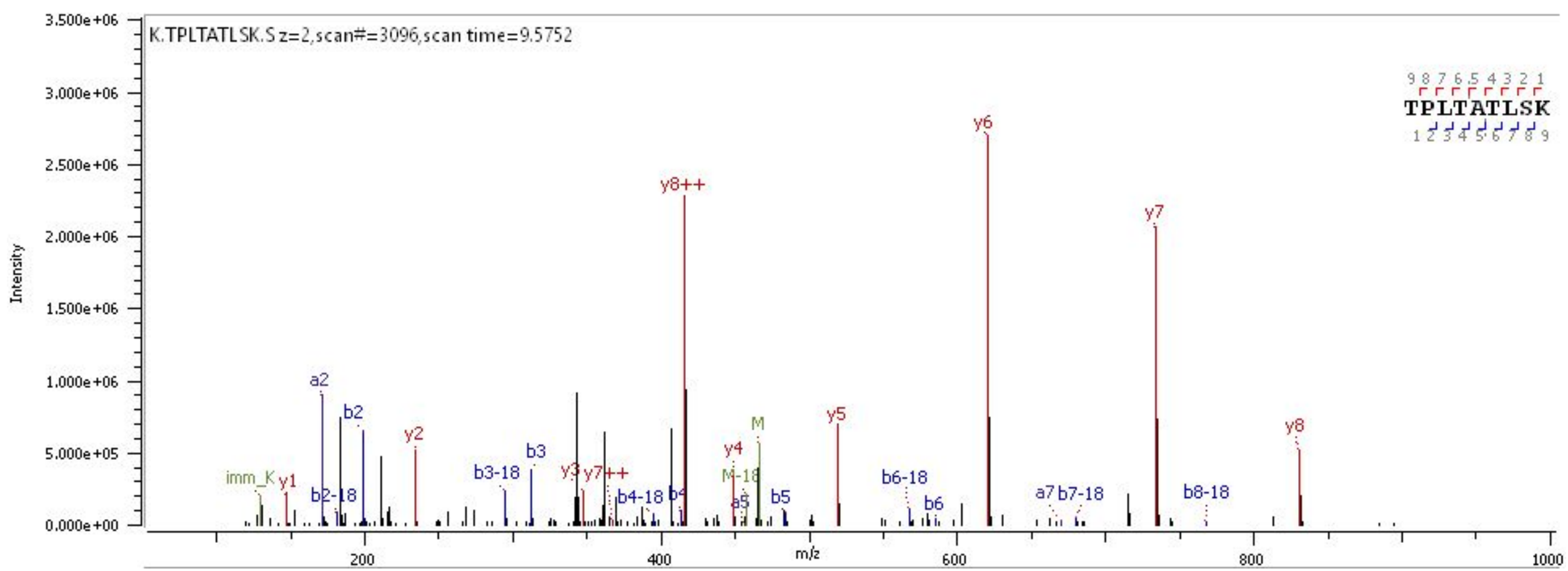

Sequence = WLQGSQELPR, z=2, retention time=10.2105

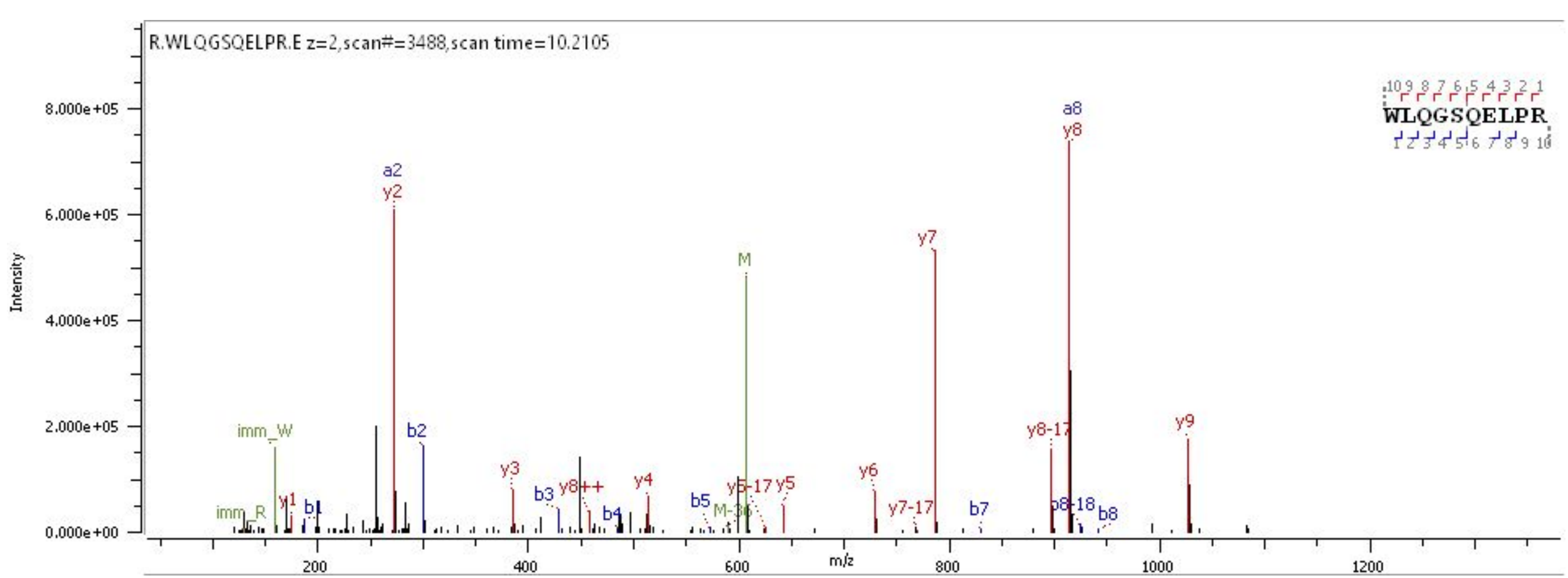

Sequence = EKYLTWASR, z=3, retention time=12.6838

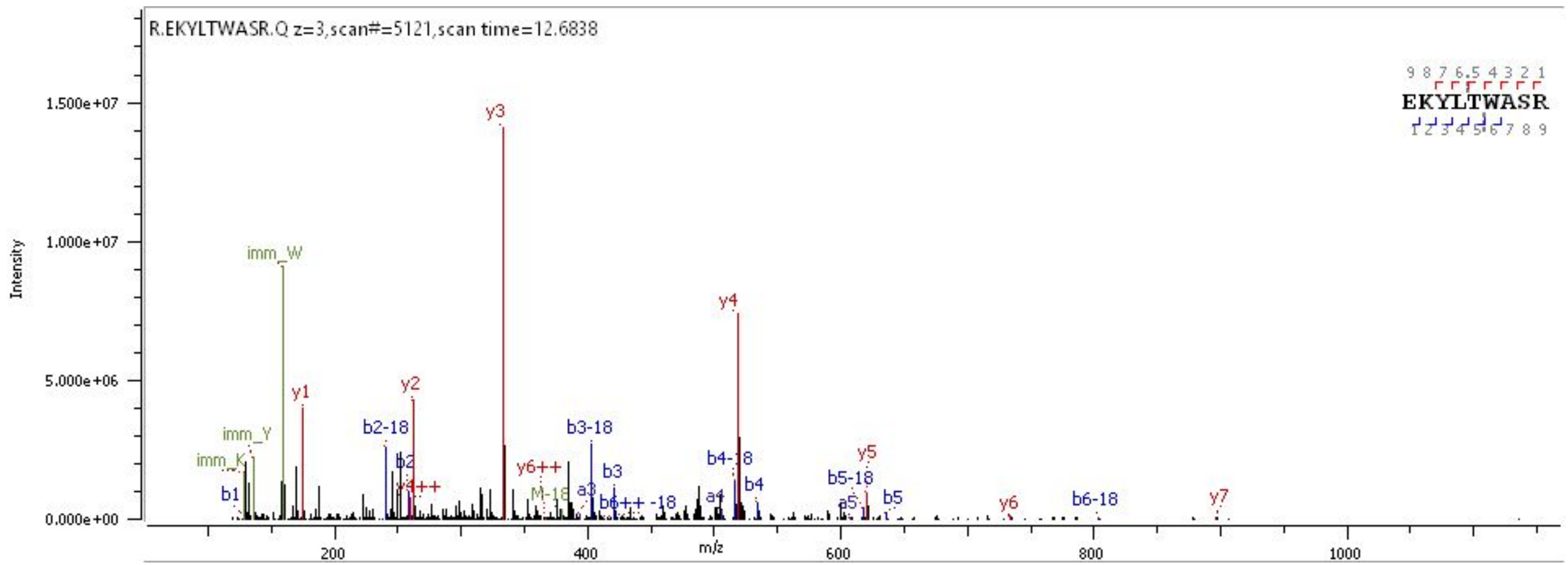

Sequence = QEPSQGTTTFAVTSILR, z=2, retention time=19.8534

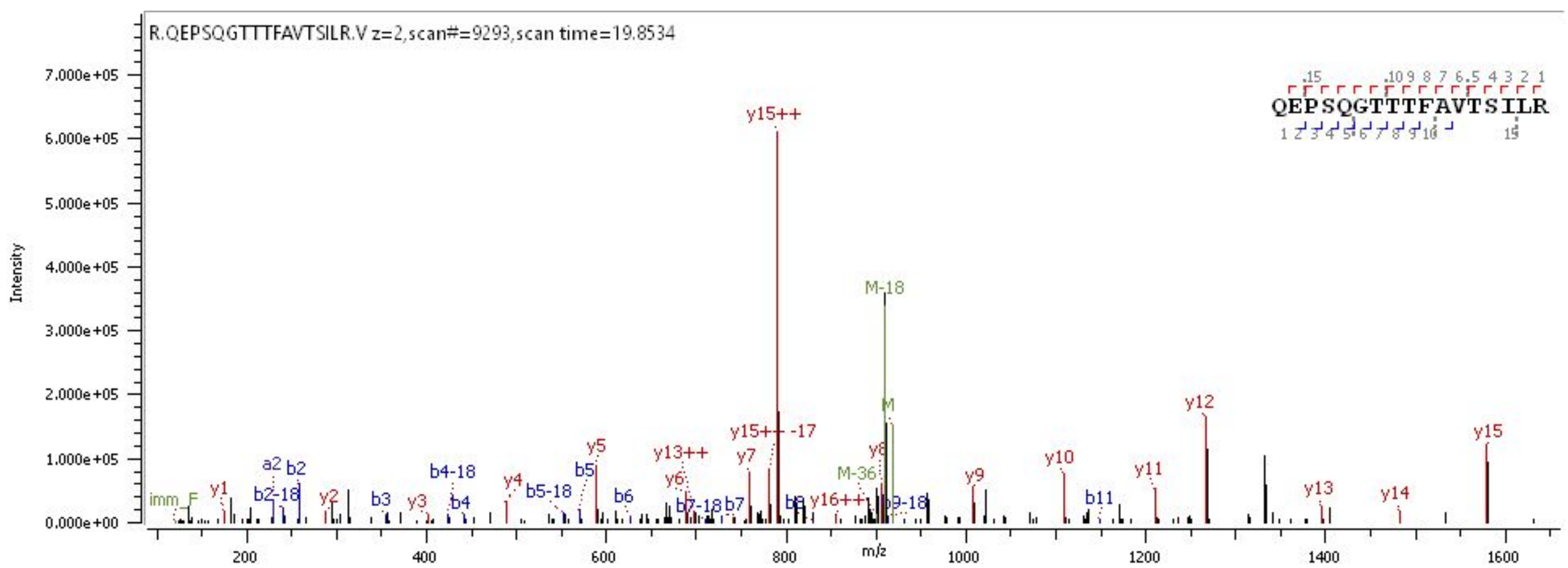

Sequence = KGDTFSC[+57]MVGHEALPLAFTQK, z=4, retention time=13.8145

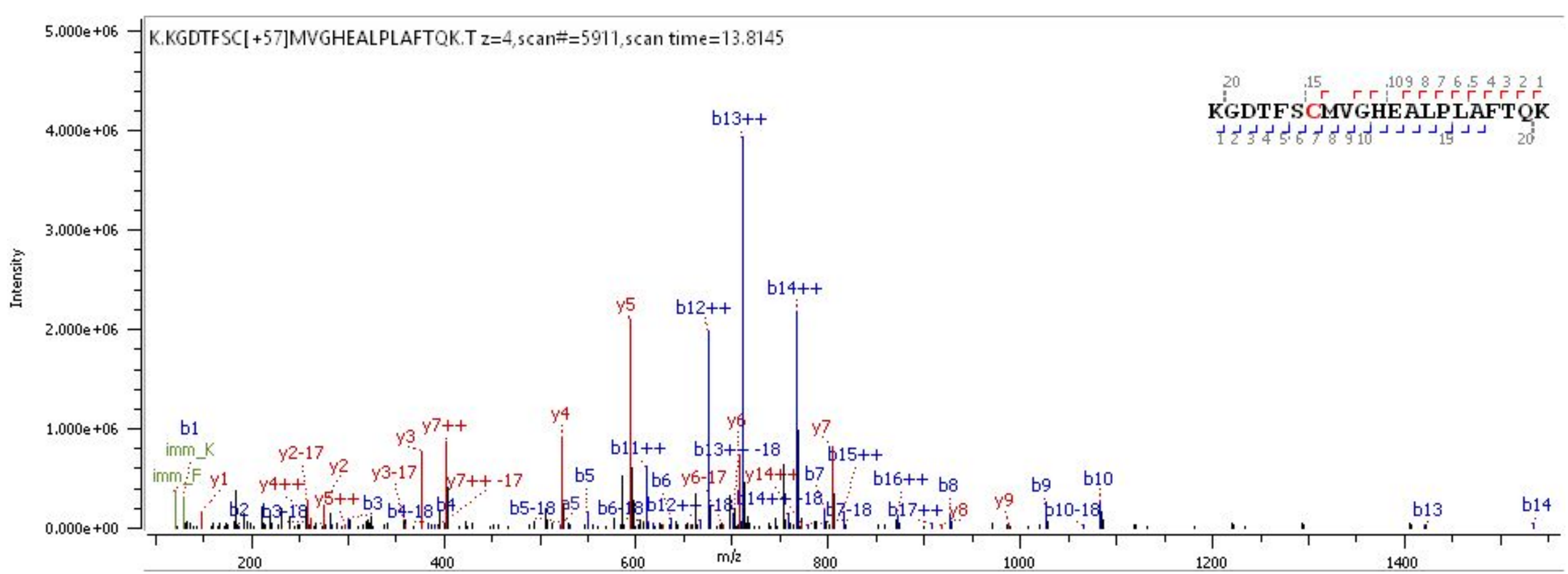

Supplement: Supplementary file 2 [file ja6c12113_si_004.pdf]
