# Supplementary material for: Intricate Proteoform Heterogeneity in the Tailpiece of IgA within and across Its Circulatory and Secretory Configurations
Source: J Am Chem Soc. 2026 Oct 21;148(43):46733–46. doi: 10.1021/jacs.6c12113 (PMC13741831; doi:10.1021/jacs.6c12113)
Supplement: Supplementary file 3 [file ja6c12113_si_006.pdf]

Supplementary Materials

Intricate proteoform heterogeneity in the tailpiece of IgA within and across its circulatory and secretory configurations

Weiwei Wang[1,2,3], Amber D. Rolland[1,2], Gestur Vidarsson[1,2,4], Johannes B van Goudoever[5], Karli R. Reiding[1,2], Albert Bondt[1,2], Albert J.R. Heck[1,2]*

[1] Biomolecular Mass Spectrometry and Proteomics, Bijvoet Center for Biomolecular Research and Utrecht Institute for Pharmaceutical Sciences, Utrecht University, Padualaan 8, 3584 CH Utrecht, The Netherlands.

[2] Netherlands Proteomics Center, Padualaan 8, 3584 CH Utrecht, The Netherlands.

[3]School of Pharmaceutical Science, Shanghai Jiao Tong University, 800 Dongchuan Road, 200240 Shanghai, People's Republic of China.

[4] Sanquin Research and Landsteiner Laboratory, Department of Experimental Immunohematology, Amsterdam University Medical Center, 1066 CX Amsterdam, The Netherlands

[5] Amsterdam University Medical Center, Emma Children's Hospital, Amsterdam Reproduction & Development Research Institute, Department of Pediatrics, Meibergdreef 9, 1105 AZ Amsterdam, The Netherlands

*Correspondence: Prof. dr. Albert J.R. Heck, email: a.j.r.heck@uu.nl

Table S1. Overview of IgA1 and IgA2 *N*-glycosylation amino acid site numbering. Multiple immunoglobulin sequence numbering conventions are used in parallel in the literature. The most used convention is based on early immunoglobulin sequencing data, whereas UniProt numbering relies on conserved full-length sequences and IMGT numbering is based on domain alignment and homology[1]. In this work, we adopted the conventional literature-based numbering system. *IgA2 exhibits an allotype-dependent difference in *N*-glycosylation at Asn211, which is present in IgA2m2 but absent in IgA2m1.

	**Conventional literature**	**UniProt**	**IMGT-domain**
IgA1	263	144	CH2-20
IgA1	459	340	CHS-7
IgA2	166	47	CH1-45.2
IgA2*	211	92	CH1-114
IgA2	263	131	CH2-20
IgA2	337	205	CH2-120
IgA2	459	327	CHS-7

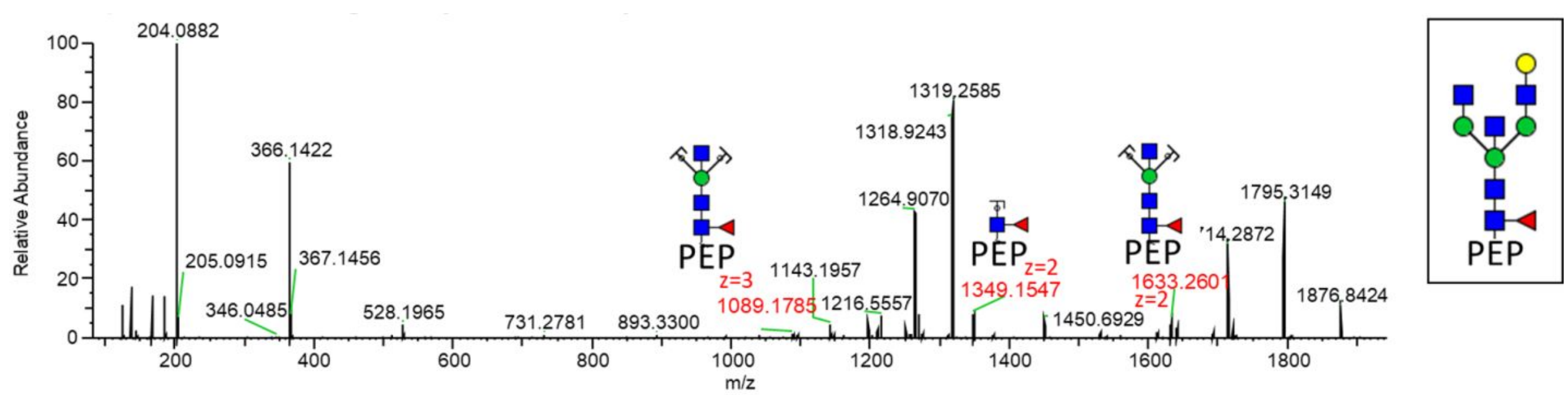

Figure S1. Diagnostic evidence for bisecting GlcNAc and core fucosylation on the IgA tailpiece glycopeptide. Representative HCD-MS2 spectrum of the donor 4 milk IgA tailpiece glycopeptide LAGKPTHVNVSVVMAEVDGTCY carrying the N5H4F1 glycoform (precursor m/z 1039.9661, z = 3; RT, 31.22 min). The diagnostic Y ion for bisecting GlcNAc, peptide–GlcNAc–GlcNAc–Man–GlcNAc, was observed at two complementary charge states: m/z 1089.1785 (z = 3) and m/z 1633.2601 (z = 2). In addition, the diagnostic Y ion for core fucosylation, peptide–GlcNAc–Fuc, was observed at m/z 1349.1547 (z = 2). Together, these diagnostic ions support the assignment of bisecting GlcNAc and core fucosylation in the N5H4F1 glycoform.

Rationale for sialic acid linkage assignment.

We used the IgA tailpiece glycopeptide LAGKPTHVNVSVVMAEVDGTCY carrying the disialylated N4H5S2 glycoform to determine the sialic acid linkage (α-2,3 vs. α-2,6) present on this glycopeptide. For this disialylated N4H5S2 glycoform, each sialic acid could be either α-2,3- or α-2,6-linked and may reside on either the 3-branch or the 6-branch of the diantennary glycan. As both branches are sialylated in this glycoform, four theoretical linkage combinations exist: α-2,6+α-2,6, α-2,3+α-2,6, α-2,6+α-2,3, and α-2,3+α-2,3. Sialic acid linkage type was determined from the relative intensities of a set of characteristic low-mass fragment ions generated during HCD fragmentation, corresponding to HexNAc (m/z 204.0867), NeuAc−H2O (m/z 274.0921), NeuAc (m/z 292.1025), and HexNAc-Hex (m/z 366.1397). The intensity ratio was calculated as (NeuAc−H2O + NeuAc)/(HexNAc + NeuAc−H2O + NeuAc + HexNAc-Hex)[2]: because α-2,3- and α-2,6-linked sialic acids differ in their propensity to cleave independently during HCD fragmentation, a smaller ratio indicates a higher proportion of α-2,6 linkage, while a larger ratio indicates a higher proportion of α-2,3 linkage. In addition, we have previously established, for glycopeptides sharing the same peptide backbone[3], that differences in glycan structure shift retention time in a consistent, structure-dependent direction. Accordingly, the two "homo" combinations (α-2,6+α-2,6 and α-2,3+α-2,3, in which both sialic acids share the same linkage) were expected to elute at the two extremes of the retention time window, while the two "hetero" combinations (one α-2,3- and one α-2,6-linked sialic acid) were expected to elute in between.

For recombinant monomeric IgA (rIgA/mIgA, HEK293-expressed), the N4H5S2 glycopeptide was resolved into four chromatographic peaks by HILIC-LC-MS/MS (Figure S2 A), consistent with the four theoretical combinations above. Ordered by retention time, Peak 1 (RT 72.34) and Peak 4 (RT 76.45) exhibited the lowest and highest fragment intensity ratios (0.469 and 0.624, respectively) and were therefore assigned as the homo linkage forms α-2,6+α-2,6 and α-2,3+α-2,3; Peak 2 (RT 73.29, ratio 0.508) and Peak 3 (RT 75.71, ratio 0.531) exhibited intermediate ratios and were assigned as hetero (mixed α-2,3/α-2,6) forms, in agreement with the expected elution order. Endogenous human IgA (colostrum-derived) yielded only a single major chromatographic peak (RT 71.92, ratio 0.489) for the same glycopeptide, which matched rIgA Peak 1 in both retention time (71.92 vs. 72.34 min) and fragment intensity ratio (0.489 vs. 0.469). We therefore conclude that the sialic acids on this tailpiece glycopeptide from endogenous IgA are predominantly α-2,6-linked (α-2,6+α-2,6), with no evidence of α-2,3 linkage. The corresponding chromatograms and MS2 spectra are shown in Figure S2.

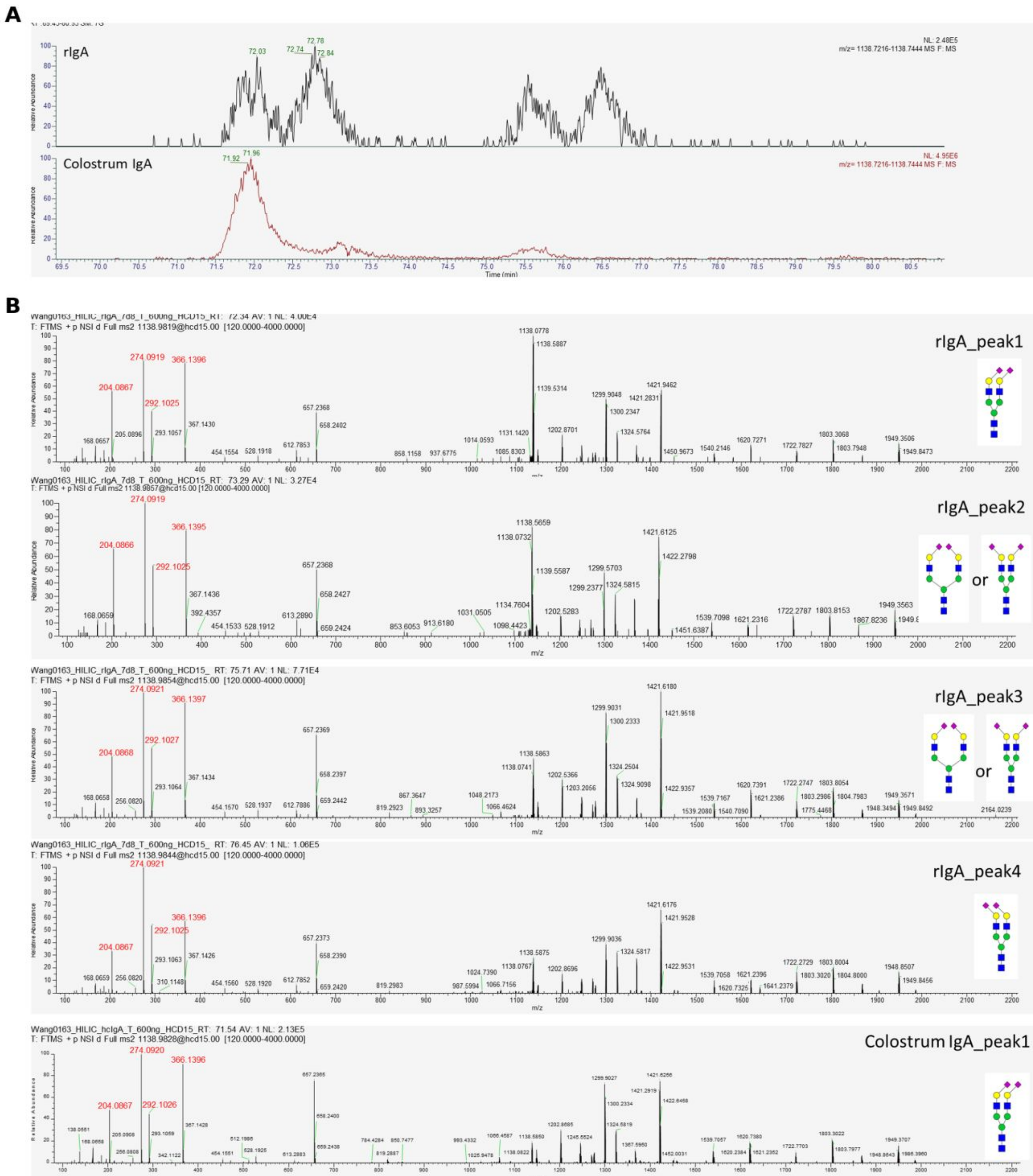

Figure S2. Chromatographic and MS2 data supporting the assignment of sialic acid linkage on the IgA tailpiece glycopeptide LAGKPTHVNVSVVMAEVDGTCY carrying the disialylated N4H5S2 glycoform. (A) Extracted ion chromatograms (monoisotopic precursor m/z 1138.7338, extraction window m/z 1138.7216–1138.7444) for recombinant monomeric IgA (rIgA, HEK293-expressed; top, black) and endogenous human IgA (colostrum IgA; bottom, red). (B) MS2 spectra of the four rIgA chromatographic peaks (top to bottom: Peak 1, RT 72.34; Peak 2, RT 73.29; Peak 3, RT 75.71; Peak 4, RT 76.45) and of the single major chromatographic peak observed for colostrum IgA (RT 71.92), with the corresponding sialic acid linkage assignment depicted alongside each spectrum.

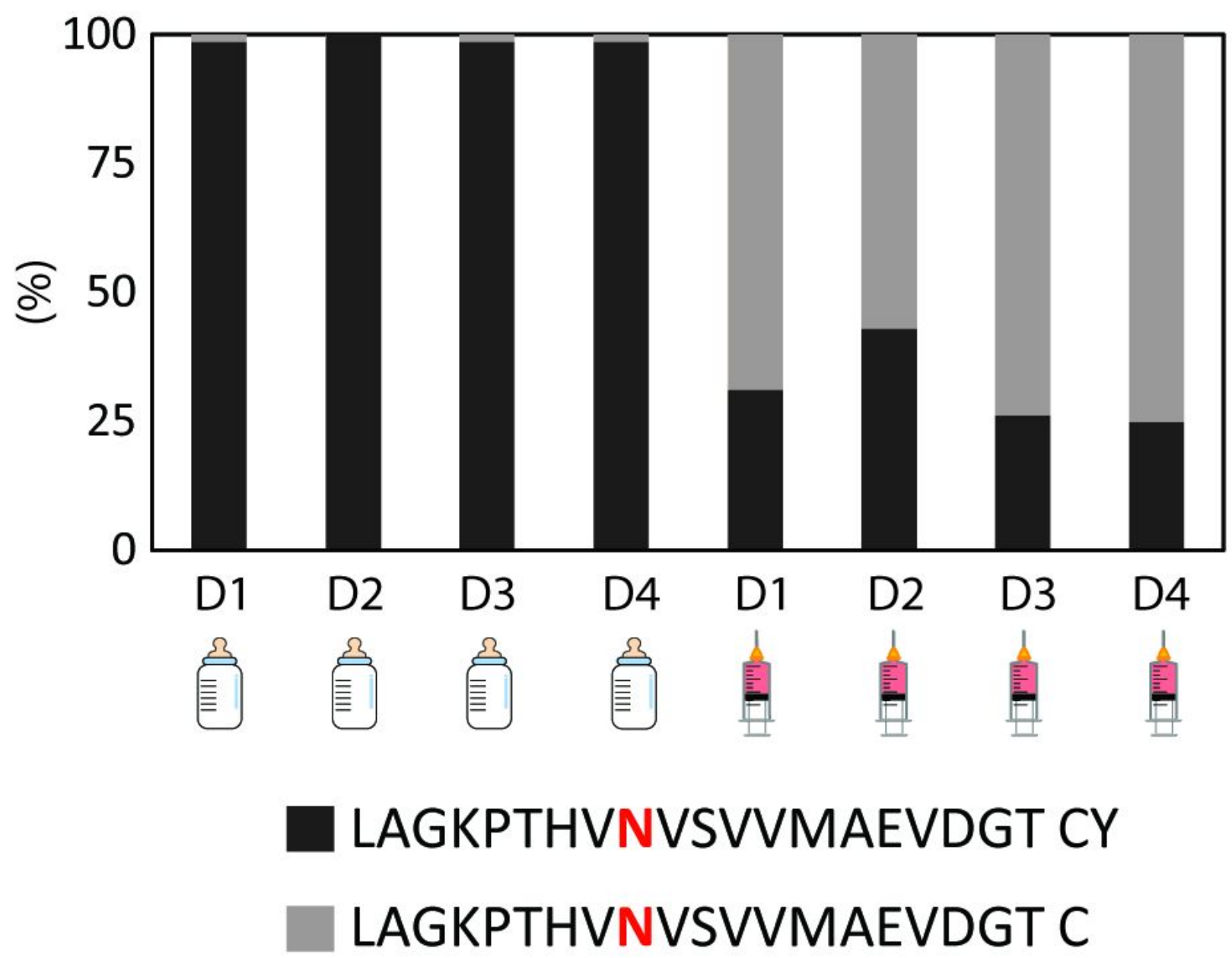

Figure S3. Distribution of IgA tailpiece sequence variants in paired breastmilk and serum of Donors 1-4. Two tryptic peptides containing the IgA tailpiece sequence but differing in the presence/absence of the C-terminal tyrosine residue were detected in glycoproteomics analyses of breastmilk- and serum-derived IgA of Donors 1-4. The relative abundances of full-length Tyr472+ tailpieces (dark grey) and truncated Tyr472− tailpiece sequence variants terminating with Cys471 (light grey) were determined based on the quantified glycopeptide areas. In milk-derived IgA, Tyr472+ tailpieces were present almost exclusively. In contrast, both variants were detected in serum-derived IgA, with 25–42% of tailpieces retaining the C-terminal Tyr472 and the majority (58–75%) of tailpieces lacking the C-terminal Tyr.

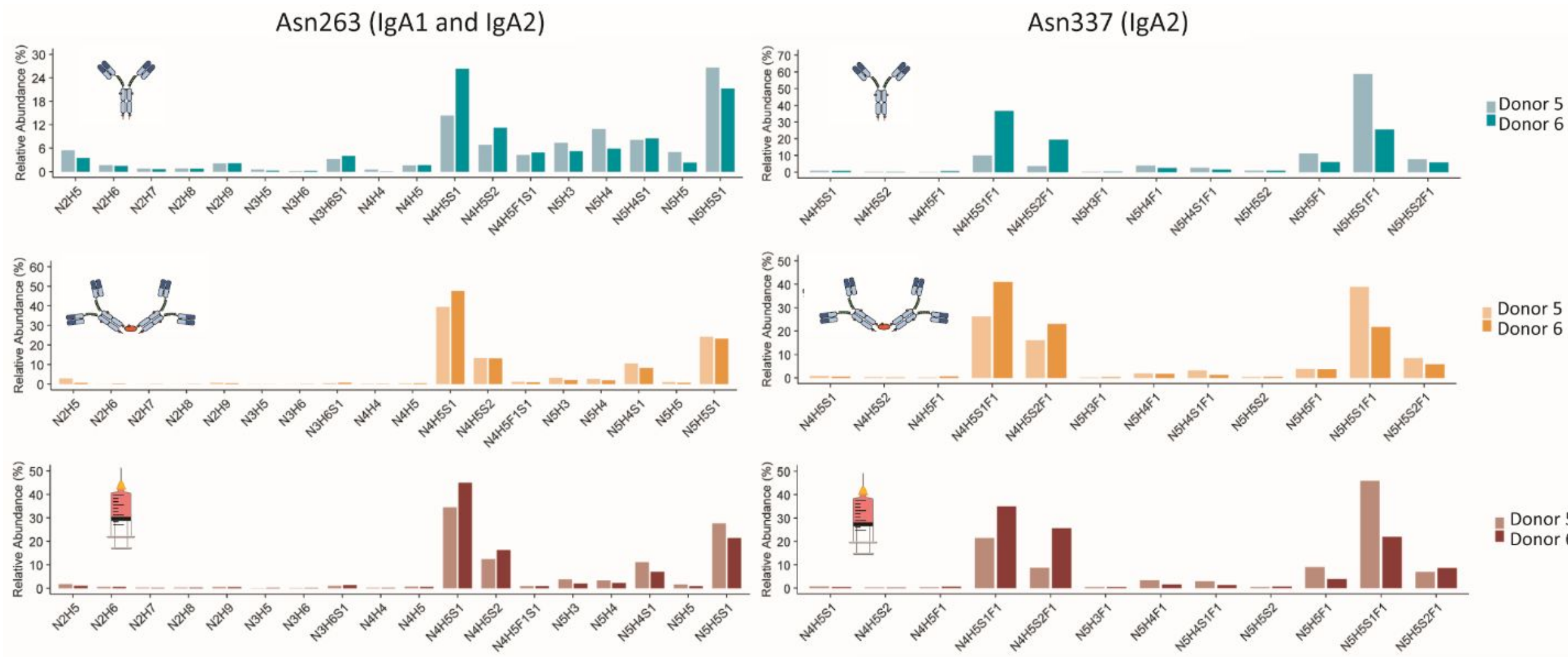

Figure S4. *N*-glycosylation signatures in the CH2 domain of monomeric and dimeric serum-derived IgA. The CH2 domain of IgA contains two major *N*-glycosylation sites, one of which is fully conserved across all subclasses/allotypes (corresponding to Asn263 in IgA1 and Asn 337 in IgA2; left) while the other (Asn337; right) is exclusive to the IgA2 subclass. CH2 domain site-specific glycosylation profiles shown were determined by analysis of the glycopeptides generated from digestion of monomeric IgA (top row), J-coupled dimeric IgA (middle), and total circulatory IgA (bottom row) purified from the serum of two healthy individuals (Donors 5 and 6). Glycan signatures were found to be nearly identical between mIgA and dIgA for the same CH2 site, indicating that *N*-glycosylation patterns in this domain are largely independent of the molecular form of IgA.

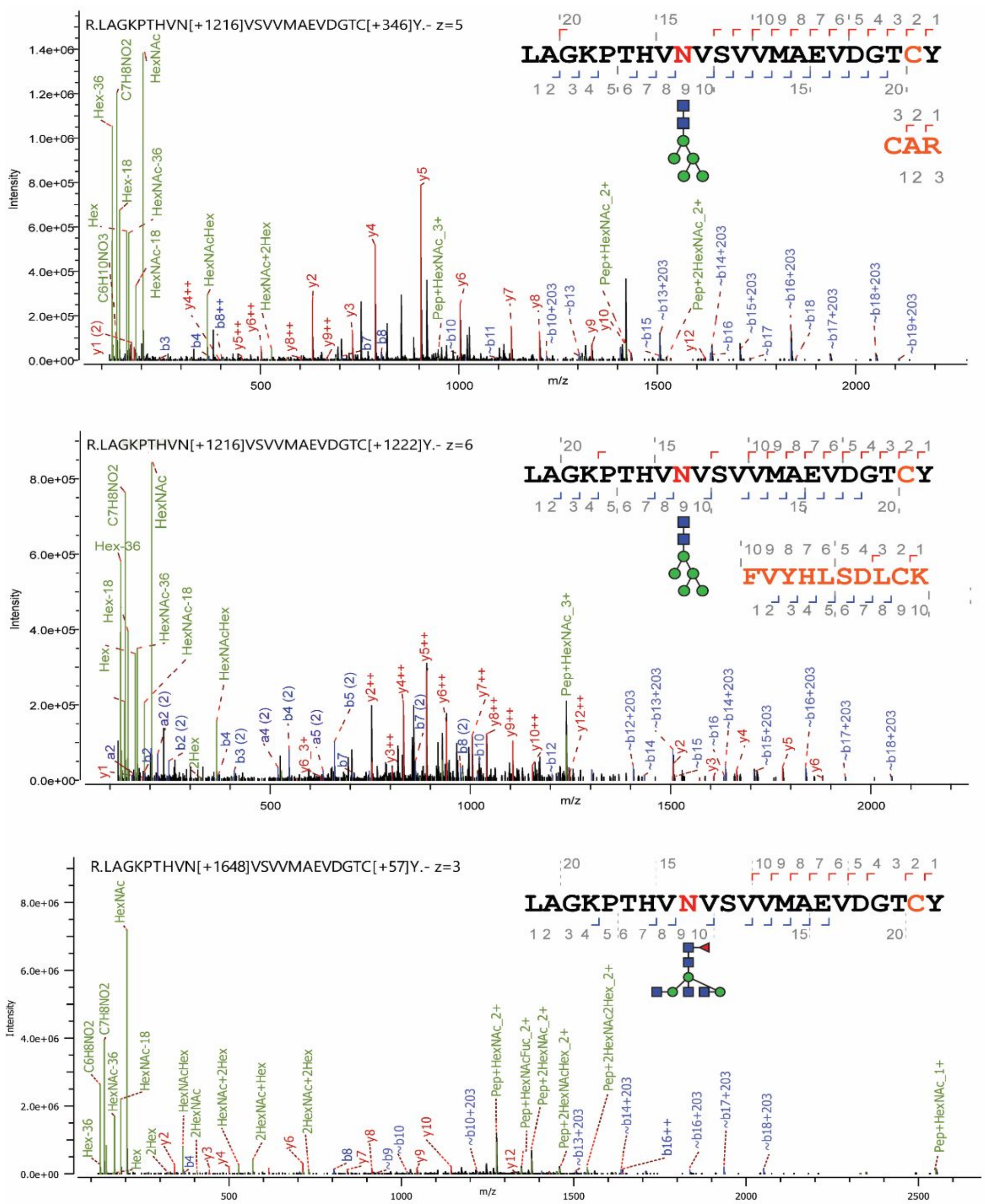

Figure S5. HCD-MS2 characterization of the three IgA tailpiece Cys471 states in human colostrum SIgA. Representative annotated HCD-MS2 fragmentation spectra of the Cys471–Cys37 disulfide-linked glycopeptide (top), Cys471–Cys91 disulfide-linked glycopeptide (middle), and free Cys471-containing glycopeptide (bottom), together with their assigned glycan compositions. For both Cys471–Cys37 and Cys471–Cys91 disulfide-linked glycopeptides, backbone b- and y-ions derived from the IgA tailpiece peptide, as well as b(2) and y(2) ions derived from the J-chain peptides, were observed, supporting the assignment of the corresponding disulfide linkages. In addition, glycan-derived oxonium ions and peptide ions retaining one or two HexNAc residues (Pep+HexNAc and Pep+2HexNAc) supported the assigned glycan compositions.

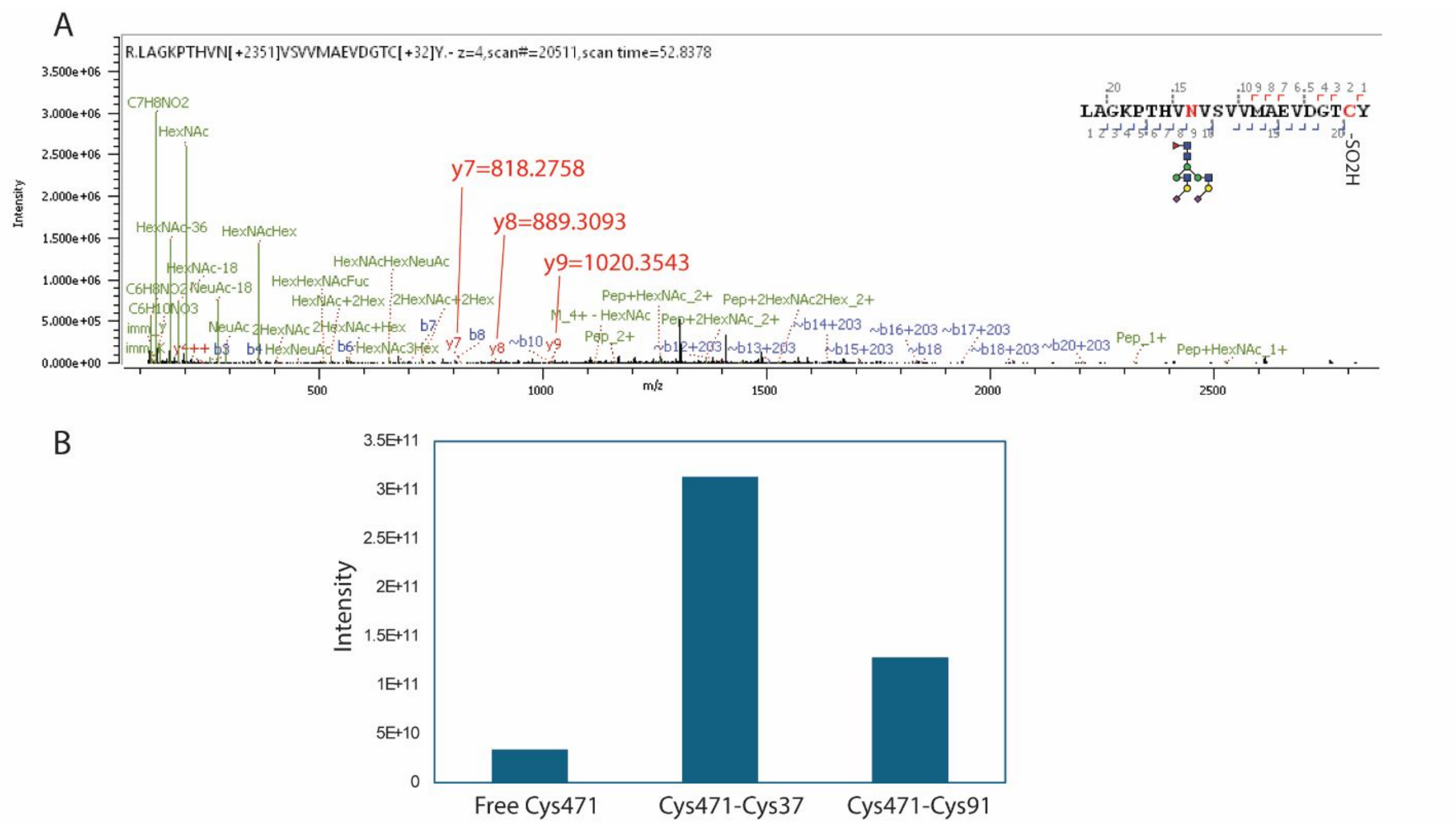

Figure S6. Signal dilution of free Cys471 due to heterogeneous peptide modifications in human colostrum SIgA.

(A) Annotated HCD MS2 fragmentation spectrum of a free Cys471 SIgA tailpiece-containing glycopeptide carrying a cysteine sulfinic acid modification at Cys471 and N4H5F1S2 glycan at Asn459 (precursor ion m/z 1169.2423, charge 4+). Tailpiece-containing glycopeptides were generated from tryptic digestion of SIgA under non-reducing conditions, with alkylation of most free Cys471 residues in unlinked tailpieces. *S*-sulfinylation of the free Cys471 shown here is just one example of the numerous other cysteine/thiol group modifications detected. (B) Summed signal intensities of all glycopeptides corresponding to each of the three Cys471 tailpiece states detected in human colostrum SIgA. Tryptic digestion of SIgA under non-reducing conditions yielded glycopeptides both for tailpieces disulfide-linked to the J-chain (Cys471–Cys37, Cys471–Cys91) as well as free unlinked tailpieces (Free Cys471).. In the former two species, Cys471 forms a disulfide bond with cysteines in the J-chain (Cys37 or Cys91) and is therefore occupied. In contrast, Cys471 in the Free Cys471 species is predominantly present in a free thiol state, although a minor fraction carries oxidative modifications such as *S*-sulfinylation. Together with the intrinsic glycan heterogeneity of the tailpiece glycopeptide, these peptide-level modifications substantially disperse the signal across multiple glycopeptide forms, thereby diluting the detectable signal of Free Cys471. As a consequence, the total observed signal of Free Cys471 appears lower than that of the disulfide-linked species (Cys471–Cys37 and Cys471–Cys91).

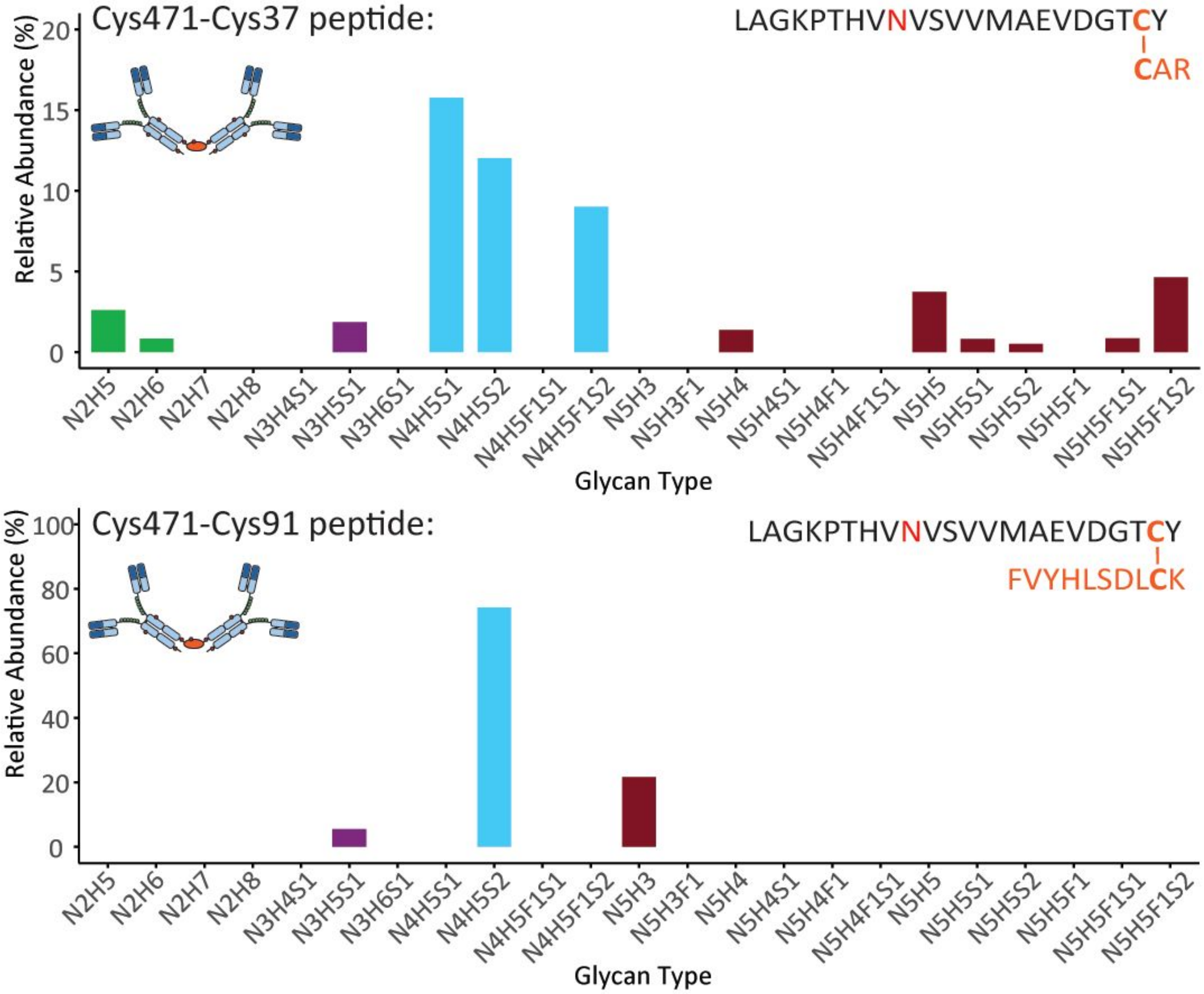

Figure S7. Glycan signatures in tailpiece-containing glycopeptides generated from the serum dIgA of Donor 5 by digestion under non-reducing conditions. The serum-derived dIgA of Donor 5 was subjected to digestion under non-reducing conditions using the same protocol applied for SIgA. Theoretically, three types of tailpiece-containing glycopeptides could be generated based on the redox state of Cys471 and disulfide connectivity with the J-chain: Cys471–Cys37, Cys471–Cys91, and free Cys471. No signal corresponding to the free Cys471 tailpiece-containing glycopeptides was detected in serum dIgA, which is most likely attributed to the limited amount of dIgA starting material overall and further dilution and dispersal of any measurable signal by heterogeneous modifications of Cys471. We identified Cys471–Cys37 and Cys471–Cys91 disulfide-linked glycopeptides and characterized their glycan signatures based on retention times, using the corresponding SIgA glycopeptides as references. Distinct glycan profiles could be observed for these two tailpiece configurations involved in distinct disulfide linkages with the J-chain, consistent with the heterogeneity patterns observed for non-reduced SIgA tailpiece glycopeptides.

Supplementary References

[1] Plomp R, Bondt A, de Haan N, Rombouts Y, Wuhrer M. Recent Advances in Clinical Glycoproteomics of Immunoglobulins (Igs). Mol Cell Proteomics. 2016 Jul;15(7):2217-28.

[2] Maliepaard JCL, Damen JMA, Boons GJPH, Reiding KR. Glycoproteomics-Compatible MS/MS-Based Quantification of Glycopeptide Isomers. Anal Chem. 2023 Jun 27;95(25):9605-9614.

[3] Wang W, Maliepaard JCL, Damelang T, Vidarsson G, Heck AJR, Reiding KR. Human IgG Subclasses Differ in the Structural Elements of Their N-Glycosylation. ACS Cent Sci. 2024 Oct 30;10(11):2048-2058.
